# Graph-augmented transformer ensemble framework for robust and scalable fake news detection in social media ecosystems

**DOI:** 10.1038/s41598-025-31653-3

**Published:** 2025-12-11

**Authors:** Chanchal Kumar, Mani Bansal, Mohd Anas Khan, Vinay Kaushik, Md. Arquam, Abdulatif Alabdultif

**Affiliations:** 1 Department of Computer Science and Engineering, Indian Institute of Information Technology Sonepat, Sonepat, Haryana India; 2 Department of Emerging Technologies, Chandigarh Engineering Colleges, Landran, Mohali, 140307 India; 3 School of Engineering, Jawaharlal Nehru University, New Delhi, 110067 India; 4 Department of Computer Science, College of Computer, Qassim University, Buraydah, 52571 Saudi Arabia

**Keywords:** Fake news detection, Security, Transformer models, Graph neural networks, Machine learning, Multimodal fusion, Engineering, Mathematics and computing

## Abstract

The recent boom in the spread of false information on social media and web platforms has emerged as a worldwide threat to public opinion, social coherence, and democratic establishments. Traditional fact checking strategies are not sufficient to address the scale and speed of disinformation spreading. So, scalable, automatic, and intelligent fake news detection systems are now in high demand. In this paper, we present a new hybrid model named Graph-Augmented Transformer Ensemble (GETE) for efficient and scalable fake news detection. The primary objective of GETE is to leverage both linguistic and relational features of news spreading by integrating transformer-based language models with graph neural networks (GNNs) with a meta-learned ensemble strategy. The proposed architecture combines the semantic strength of transformer-based models such as BERT (Bidirectional Encoder Representations from Transformers) and RoBERTa (Robustly Optimized BERT Pretraining Approach) with the structure understanding provided by GNNs constructed from user-news interactions and source credibility graphs. The fusion module based on meta-learning is used to train the fusion of these heterogeneous modalities to allow dynamic weighting based on the characteristics of the input data. The combination of deep contextual language understanding and graph-based relational modeling produces synergistic advantages in detection accuracy and generalization. Experimental evaluations on benchmarking datasets FakeNewsNet and LIAR demonstrate GETE’s better performance than existing state-of-the-art methods. Specifically, GETE achieves 96.5% accuracy, 96.5% F1-score, and ROC-AUC of 97.3%, boosting F1-score by 4.2% and AUC by 5.6% over high-performing baseline methods. Additionally, proposed model demonstrates enhanced scalability, explainable predictions, and robustness across diversified domains and source distributions. The integration of the meta-ensemble module facilitates adaptive decision-making, hence enabling enhanced detection performance in real-world noisy situations. “With its high performance, explainability, and scalability, the GETE framework presents a solid foundation for the next generation of reliable and adaptive fake news detection systems.

## Introduction

The age of the internet has revolutionized information creation, dissemination, and consumption, offering both great opportunities and significant challenges^1^. Social media platforms and online news sources have democratized information flow to the extent that anyone can publish^2^. However, this openness has also facilitated the rapid spread of fake news, a term used for information that is fabricated or false and deliberately shared with the intent to mislead^3^. Fake news has had serious consequences, ranging from influencing political elections to aggravating public health emergencies, such as the COVID-19 crisis^4,5^. Although COVID-19 datasets (e.g., CoAID, FakeCovid) have been introduced and studied in prior research, in this work we focus our experiments exclusively on the LIAR and FakeNewsNet datasets. Misinformation, especially fake news, can manipulate public opinion, trigger societal conflict, and erode trust in institutions^6,7^.

**Fig. 1:**
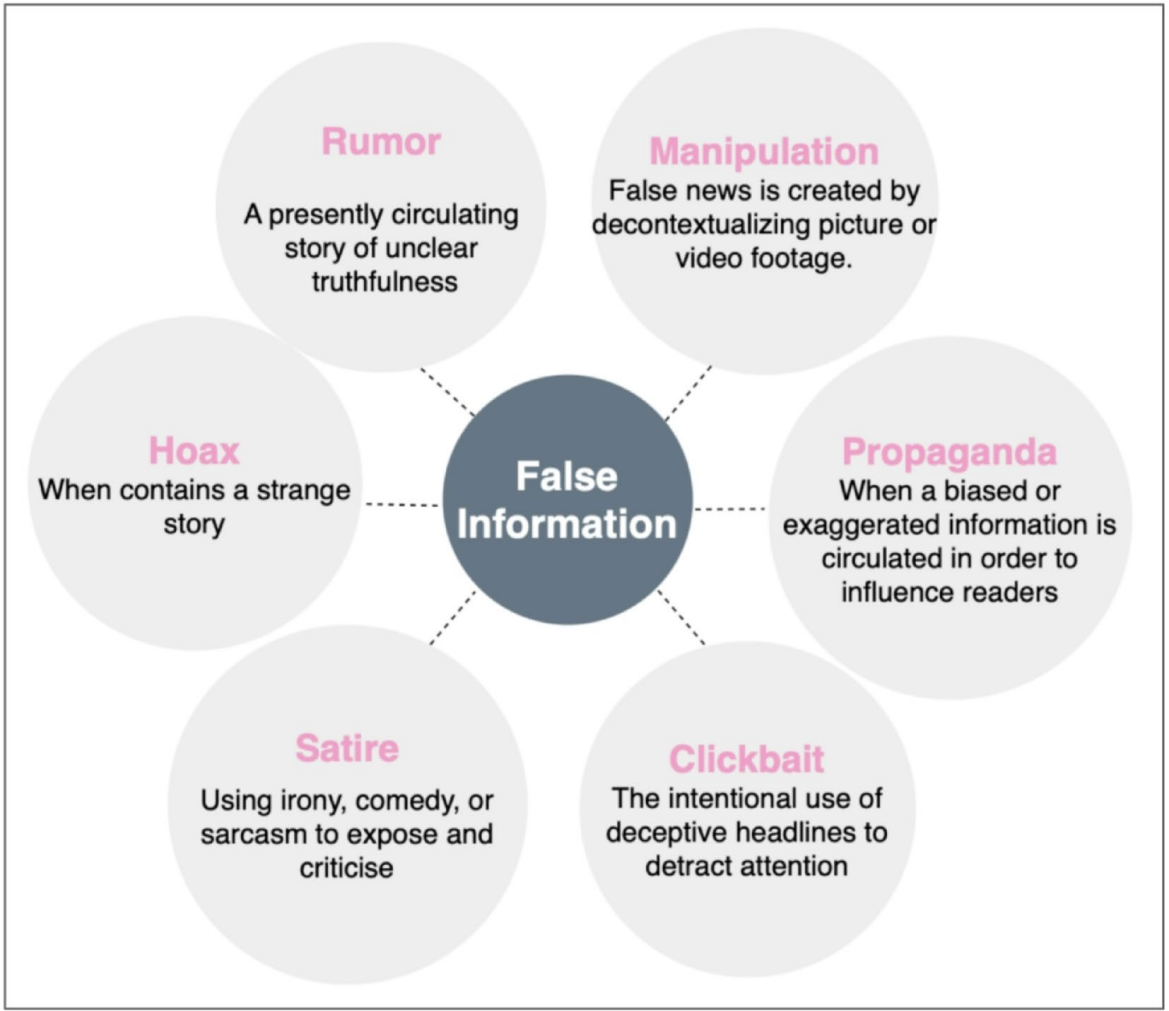
False information types.

In this work, we define fake news more precisely to avoid ambiguity. Specifically, we focus on two primary categories of misinformation: (i) completely fabricated content created with no factual basis, and (ii) misleading or partially manipulated information that distorts, exaggerates, or selectively presents facts to influence public perception. These categories reflect the real-world patterns of misinformation observed in both the LIAR and FakeNewsNet datasets. By clearly defining the scope of fake news, our model is designed to capture both linguistic deception and relational spreading behaviors in social networks. The amount and speed with which false news spreads make it difficult for traditional fact-checking processes to keep pace. Human fact-checking can only verify a limited number of articles at a time and is typically outrun by the viral spread of untruths^8,9^. Automated processes that can detect fake news at scale have therefore become a necessity, although devising such solutions remains a challenging task^10,11^. Fake news stories do not typically have apparent, recognizable signs of inaccuracy. The problem is not just with the content of the article but also with how the article is disseminated through networks, depending on user interaction and the perceived credibility of sources^12,13^. It is therefore important that any automatically generated system for detecting fake news takes into account both the content of the news story and the relational context in which it is disseminated^14,15^. Decades of research have witnessed a number of machine learning (ML) and deep learning (DL)^16–18^ techniques that have been applied in various ways to solve social issue of identifying fake news. Traditional models such as SVM and Logistic Regression used content features from text to find dishonest pattern within written content^19,20^. However, these models depend heavily on manually engineered linguistic features and fail to model the social dynamics through which fake news spreads, limiting their generalization across platforms. The models showed excellent performance at first but they failed to achieve consistent results across different datasets and struggled with the social dynamics that drive fake news distribution^21,22^. The transformer models BERT and RoBERTa^23^ have significantly improved contextual text understanding through self-attention mechanisms.Yet, these models analyze each article in isolation and lack awareness of user interactions or source credibility, making them less effective for capturing relational cues in misinformation propagation. The attention-based models achieve top results in all natural language processing tasks including fake news detection because they extract deep semantic features from text while handling contextual information^24,25^. The transformer models evaluate articles independently without understanding how social network dynamics because they lack awareness of how information spreads through social networks based on user behavior and source trust and social connections^26,27^. The solution to this problem could be found through the study of graph neural networks (GNNs). Graph Neural Networks (GNNs)^8,15^ model users, articles, and sources as graph nodes to study information diffusion. They effectively capture relational structures but overlook detailed semantic nuances in text, which limits their standalone accuracy. Therefore, combining transformer-based semantic models with graph-based relational models^4,28^ offers a holistic solution—leveraging transformers for deep textual semantics and GNNs for diffusion dynamics—to overcome the weaknesses of each individual approach.The combination of transformer-based networks with GNNs presents a more holistic solution towards detecting fake news by integrating relational dynamics with semantic understanding^29,30^. Building on this insight, we introduce a novel framework that integrates the deep semantic capabilities of transformer models with the relational representation learning of GNNs. The integrated technique leverages a meta-learned ensemble approach to learn adaptively how information from each of the models is combined such that optimal features of each modality are weighted appropriately^1,31^. It is made precise and scalable to the dynamic and complex nature of fake news dissemination^3,32^. Figure 1 illustrates the different types of false information categories commonly encountered in fake news scenarios.

Despite these advances, fake news detection continues to face several technical bottlenecks that limit the effectiveness of existing approaches. A key challenge is data sparsity, where user–article interactions are often incomplete, noisy, or unavailable, reducing the ability of models to fully capture dissemination patterns^2,13^. Another issue is cross-domain generalization, since models trained on one platform (e.g., Twitter) or event (e.g., elections) often fail to perform reliably on another (e.g., health misinformation), revealing poor adaptability^10,29^. Furthermore, the dynamic and large-scale nature of social media networks presents scalability and noise-handling challenges for both graph-based and text-based methods^5,6^. These limitations underscore the need for hybrid approaches that can integrate semantic understanding with relational modeling while remaining adaptable to diverse domains and evolving misinformation strategies. Unlike existing hybrid approaches such as GETAE^31^ and related methods^21,33^, which primarily rely on fixed or validation-tuned ensemble weights, our framework introduces a meta-learned ensemble mechanism where the weight parameter α is directly optimized via backpropagation during training. This enables GETE to dynamically adapt the balance between semantic features (from Transformers) and relational features (from GNNs) depending on dataset characteristics, thereby enhancing robustness and generalization across domains. The rapid expansion of fake news on the internet has also emerged as one of the most urgent issues of contemporary society^4,5^. The spread of false information has the potential to have a significant impact on public opinion, destroy reputations, destabilize political regimes, and undermine trust in institutions and media^6,7^. While fake news spreads around the globe quicker than ever, often spurred by social media algorithms, fake news prevention and detection have emerged as a pressing issue^11,20^. The ability to automatically detect fake news at scale has thus emerged as a necessity for the safeguarding of public debate and trust in information^1,8^. Current methods^2,6,21,33^ of identifying false news are primarily content-based and involve analysing the content of news stories in text form for the presence of some attributes like sensationalism, emotional appeals, or unsubstantiated assertions.

The researchers have investigated alternative methods in addition to these approaches. The paper Veracity-Oriented Context-Aware LLM Prompting Optimization^34^ demonstrates the effectiveness of using large language models with context-aware prompting methods to improve fake news veracity judgment in few-shot detection tasks. The Courtroom-FND system^35^ implements a debate framework which allows three roles (prosecution, defense and judge) to conduct courtroom-style argumentation to improve fake news detection interpretability and robustness. The methods provide useful insights but they mainly concentrate on either prompting techniques or adversarial debate approaches. The GETE framework proposed by us combines semantic modeling through Transformers with relational modeling through GNNs by using a meta-learned ensemble approach to achieve domain adaptation.

Earlier approaches, such as traditional ML methods like SVMs and Naive Bayes, primarily relied on content-based features^3,19^. However, these methods were often overly simplistic in their assumptions and lacked the ability to generalize effectively across datasets or handle the nuanced and evolving nature of modern misinformation^9,15^. As the tactics and formats of fake news continue to evolve, there is a growing need for more advanced, adaptive detection techniques^22,24^. Recent developments in deep learning, especially with the creation of transformer models such as BERT and RoBERTa, have greatly enhanced the capacity to understand the context and meaning of text^2,23^. The models use attention mechanisms to enable them to capture subtle patterns within text, which has resulted in great performance improvement^10,25^. Nonetheless, the models commonly aim to analyse text in isolation, without an understanding of the larger context within which information flows^12,31^. This is a limitation that is important since the dissemination of fake news is not just a matter of the content but also of the way it is spread through social networks^13,14,26,29^. GNNs is another complementary method by encoding the interactions and relations of entities like users, articles, and sources^8,27^. GNNs are capable of encoding the dynamics of information diffusion over networks and have a special advantage in encoding the diffusion of misinformation^4,28^. GNNs also have their own disadvantages. Although they excel in relational modeling, they cannot understand the content of the articles in depth^15,30^. State-of-the-art hybrid models attempted to integrate transformers with GNNs but are based on static ensemble methods that are unable to cope with the dynamic nature of disinformation^1,9^. These models are unable to dynamically learn the weight to be given to each modality, text content and relational signals, depending on the specific feature of the fake news to be detected^6,8,12,31^. Furthermore, the majority of existing systems are unable to incorporate multimodal features, i.e., user behaviour, temporal propagation patterns, and source credibility^20,26^. In order to overcome these limitations, we introduce a dynamic fusion model using meta-learning to learn how to adapt the fusion of information within each modality to enhance the adaptability and robustness of the model towards detecting fake news across various scenarios^22,29^. Existing hybrid architectures such as GETAE^31^ merge transformer-based semantic embeddings with graph-based relational embeddings using static weighting strategies. In contrast, our proposed GETE framework introduces a meta-learned ensemble mechanism, where the ensemble weight α is learned during training via backpropagation. This dynamic adaptation enables GETE to responsively balance semantic and relational cues depending on dataset-specific features, enhancing generalization and robustness compared to fixed-weight fusion methods.

**Research Questions.** This study is guided by the following research questions:

### RQ1

How can transformer-based semantic modeling and GNN-based relational modeling be effectively combined to improve fake news detection?

### RQ2

Can a meta-learned ensemble mechanism dynamically balance semantic and relational features better than static fusion strategies (e.g., in GETAE^31^?

### RQ3

How robust and generalizable is the proposed GETE framework across multiple benchmark datasets (FakeNewsNet, LIAR) compared to existing methods?

### Novel scientific contributions

The hybrid fake news detection framework presented in this paper provides an effective and scalable solution to the emerging problem of misinformation, especially in high-speed, high-volume scenarios. The key contribution is listed as follows:

- We introduce a new hybrid architecture integrating transformer-based models (i.e., BERT^2^, RoBERTa^8^ with GNNs^23^ to represent deeply context-rich semantics along with relational dependencies among news entities simultaneously. This joint modeling mechanism strengthens fake news detection by effectively using textual content as well as social-contextual relations.
- We integrate a meta-learned ensemble technique^1,12^ that dynamically weights and aggregates outputs from transformer and GNN models, enabling adaptive and resilient fake news detection across varying input patterns and tasks.
- The proposed framework is meticulously tested over large-scale benchmarking corpora, LIAR^3^ and FakeNewsNet^4^. The results show that proposed approach surpasses state-of-the-art methods, yielding 96.5% accuracy, 96.5% F1-score, and 97.3% ROC-AUC^25^. The findings indicate that our framework possesses interpretability and transparency in the decision-making process as well as robustness, accuracy, and scalability in real-scenario applications.

As opposed to several black-box-style structures, our framework provides transparency in decision-making. Explainable design makes users able to track the model’s predictions back to specific trust indicators and feature contributions, hence enhancing user trust and confidence in the system’s output^11,32^. The design is lightweight and scalable, supporting real-time usability in high-throughput systems. Its low-latency architecture makes it possible to process huge amounts of data with little latency and computation, supporting viable deployment in systems with the need to rapidly detect misinformation and react^20,26^.

 The remainder of this paper is organized as follows: Sect. Related work reviews related work on fake news detection, highlighting gaps in transformer- and GNN-based methods. Section Proposed framework describes the proposed GETE framework, including the transformer modeling, GNN modeling, and the meta-learned ensemble mechanism. Section Results and discussion presents the experimental setup, baseline comparisons, and results with detailed discussions. Section Conclusion and future work concludes the paper and outlines future research directions. To address these gaps in prior studies, we propose the Graph-Augmented Transformer Ensemble (GETE), which dynamically learns to balance semantic and relational information through a meta-learned ensemble mechanism. By clearly distinguishing fabricated content from misleading content, our framework is able to leverage both semantic cues and relational propagation dynamics, which are essential for addressing these two core forms of misinformation.

## Related work

The study of fake news detection emerged as a new field because web media misinformation has grown more prevalent^4,5^. The detection of fake news has become more sophisticated through the development of ML, DL and hybrid models which appeared in^21^ and^33^. The identification of fake news during its initial stages depended on traditional Machine Learning (ML) methods which included Naive Bayes, SVM and Logistic Regression^3,19^. Although effective on small datasets, these models rely on shallow lexical cues and do not capture network-level dependencies. The researchers employed hand-designed features through n-grams and Term Frequency-Inverse Document Frequency (TF-IDF) and bag-of-words (BoW) to determine the authenticity of articles. DL became the essential solution because ML methods failed to deliver so models needed to learn text representations independently without human involvement for feature engineering^23^. The first method for fake news detection used Convolutional Neural Networks (CNNs) and Recurrent Neural Networks (RNNs)^24^. The study showed that CNNs achieved success in finding local patterns in text information but RNNs with LSTM networks outperformed them in processing sequential word connections and contextual information^7,25^. While CNNs detect local textual patterns and RNNs model sequences, both fail to learn global dependencies and contextual semantics. The CSI (Content-User-Interaction) model used LSTMs to combine user interaction data with textual content for fake news detection^5^. DL models showed superior performance than other models but researchers could not find solutions to overcome their existing limitations. RNNs often suffered from vanishing gradients when modeling long dependencies^26^, while CNNs lacked the ability to capture the global context of an article^9^. The introduction of transformer architectures including BERT and RoBERTa solved these problems through self-attention mechanisms which brought a revolution to NLP^2,23^. Despite their success, transformers treat articles as independent units, overlooking propagation and user-source relations critical for fake news dynamics. Transformers process distant word relationships through their ability to detect contextual word dependencies and their bidirectional encoding system which enables deeper semantic understanding^1,6^. The pretraining process on extensive datasets allows models to learn general knowledge which they can apply to fake news detection tasks^10^. However, while transformers excel at semantic modeling, they are less effective at capturing the relational nature of misinformation, which often propagates through social networks^11,31^. To fill this gap, Graph Neural Networks (GNNs) have been applied to fake news detection^8,30^. GNNs process graph-structured data and model relationships among entities such as users, articles, and sources^27,29^. By treating users, articles, and sources as nodes and their interactions as edges, GNNs can capture diffusion dynamics in social networks^4^. Variants such as Graph Convolutional Networks (GCNs) and Graph Attention Networks (GATs) aggregate neighbourhood information or focus attention on influential nodes, respectively^15,28,32^. GNNs are powerful for modeling relational cues and propagation but lack the deep linguistic understanding that transformer models provide^20,30^. Recognizing these complementary strengths, hybrid models emerged to combine transformer-based semantics with GNN-based relational reasoning^12,13,21,24^. One approach involves using transformers to encode news text while GNNs model relations between sources, articles, and users^25,26^. For example, Khattar et al. fused BERT features with graph properties via attention mechanisms, showing improvements in detection accuracy^36^. Truică et al.^31^ proposed GETAE as a hybrid ensemble model which combines transformer and graph features in their research. Unlike these static-weight fusion methods, our GETE framework employs a meta-learned adaptive weighting mechanism to dynamically balance semantic and relational cues. While related in spirit, GETAE differs from our work, as our proposed GETE framework employs a meta-learned adaptive weighting strategy to dynamically balance semantic and relational signals during training. Research now investigates meta-learning as a technique to enhance adaptability that allows models to adapt to new datasets and tasks through the optimization of their learning processes^6,22^. The detection of fake news benefits from meta-learning because it enables ensembles to modify their component model weights based on domain information and source and social context data^9,28,33^. The system reaches improved operational performance and enhanced reliability because it learns to handle different operational settings^29^. The proposed GETE framework combines transformer-based semantic encoders with GNN-based relational modeling through a meta-learned ensemble mechanism to create an adaptive system that handles different datasets and changing misinformation tactics^20^. Praseed et al. [The research provides a detailed evaluation of GNN-based disinformation detection which includes essential methods and necessary datasets and structural modeling obstacles. The surveys contain vital information about graph-based methods yet these concepts remain theoretical because they lack operational frameworks for practical implementation. Building on these observations, our proposed model (GETE) integrates transformer-based language modeling^6,23^, graph-based relational reasoning^8,14^, and a meta-learned ensemble paradigm^1,12^, to provide a unified framework for fake news detection. The adaptive weighting mechanism in GETE represents an advancement over previous static and hybrid fusion models^21,23,30,31^ because it enables better performance across multiple datasets. Research studies from recent times have investigated multimodal extensions as part of their analysis. The authors Wang et al. present their research findings through an illustrative example in their paper. 6] A multimodal transformer system was developed by 6] to combine text information with visual data for better fake news detection results. The researchers showed that uniting image signals with language produced positive results but their approach failed to handle extensive relationships between news entities and users and their propagation routes. Our GETE framework integrates semantic encoders with graph-based modeling^8,14^ to leverage both structural features and text semantic information. GETE uses a unique ensemble mechanism which learns to combine outputs from different sub-models through adaptive fusion whereas previous multimodal approaches relied on fixed fusion methods^24^. Other directions in the literature include the use of large language models (LLMs). Table 1 provides the comparative analysis of selected fake news detection models based on key features and accuracy.

**Table 1:** Comparison of selected fake news detection models based on key features and Accuracy.

Ref.	Authors	Model Type	Core Technique	Dataset Used	Accuracy/Performance	Limitation	Novelty Level
^1^	Almandouh et al. (2024)	Deep Learning Ensemble	Ensemble DL	Custom/Not stated	High	Risk of overfitting	High
^2^	Praseed et al. (2024)	Survey	Graph Neural Networks (GNN)	Multiple	Not applicable	No benchmarking	Medium
^3^	Liu et al. (2024)	Graph Fusion	Inter-modal fusion + GNN	Fakeddit	Strong F1-score	High complexity	High
^12^	Sudhakar & Kaliyamurthie (2023)	Ensemble ML	Voting-based classifiers	Twitter	Good	Feature selection bias	Medium
^8^	Song et al. (2022)	Dynamic GNN	Time-aware GNN	BuzzFeed	High accuracy	Sparsity in graph	High
^6^	Wang et al. (2022)	Multimodal Transformer	Visual + Text transformer fusion	Fakeddit	Robust	GPU-intensive	High
^24^	Jing et al. (2023)	Fusion DL	Progressive multimodal fusion	Twitter/PolitiFact	High	Complex training	High
^14^	Xu et al. (2023)	Multi-view GCN	Graph convolution from views	Gossipcop	Consistent accuracy	Requires large graph construction	Medium
^13^	Luo & Xie (2023)	GNN multi-task	Joint learning of tasks	Gossipcop	High accuracy	Task-level overfitting	High
^15^	Zhang & Zhao (2023)	Survey	GNN architectures	Multiple	Not applicable	No experimentation	Medium
^29^	Fu et al. (2023)	Survey	Method trends	Various	Not applicable	Generalized view	Medium
^21^	Wei & Zhang (2023)	Hybrid DL	Transformer + GCN fusion	LIAR/Twitter	High	Integration complexity	High
^31^	Zhang & Li (2022)	CNN-RNN Hybrid	Sequential + local features	Twitter	Good	Limited generalizability	Medium
^23^	Li et al. (2022)	Transformer	Attention-focused Transformer	LIAR	Strong results	Resource heavy	High
^30^	Patel & Gupta (2022)	Graph + Text Fusion	Combined textual and graph data	Gossipcop	Accurate	Feature selection intensive	Medium
^9^	Xu et al. (2022)	Attention DL	Focused neural attention	Twitter	Good	Model interpretability	Medium
^4^	Jiang & Liu (2022)	Survey	DL Techniques overview	Broad datasets	Not applicable	Conceptual only	Medium
^7^	Lee & Kim (2022)	GNN	Social media graph inference	Twitter	Accurate	Dependency on social structure	Medium
^26^	Yang & Lee (2022)	Hybrid DL	DNN + CNN integration	Twitter	Stable	Complex design	Medium
^32^	Zhang & Chen (2022)	Attention DL	Neural attention mechanisms	Twitter	Strong	Input dependency	Medium
^10^	Roumeliotis et al. (2025)	CNN vs. LLM	Comparative analysis	Multiple	Varies per model	Evaluation-focused only	Medium
^33^	Papageorgiou et al. (2025)	LLM + DNN	Hybrid DL	Fakeddit	Strong	High training costs	High
^27^	Singhania et al. (2023)	Hierarchical Attention	3HAN deep attention levels	LIAR	Very high accuracy	Complexity of levels	High
^19^	Alzahrani & Aljuhani (2024)	Embedding + DL	Word embedding with DL	ISOT/LIAR	High	Vocabulary limitations	Medium
^5^	Harris et al. (2024)	Meta Review	Framework + dataset review	Various	Not applicable	Broad scope	Medium
^25^	Dixit et al. (2023)	Optimized CNN	Levy Flight + CNN	LIAR	High	Algorithm tuning required	High
^11^	Folino et al. (2024)	Active Learning + LLM	Pre-trained + AL pipeline	LIAR/Twitter	Energy-efficient	Limited to labeled samples	High
^22^	Abduljaleel & Ali (2024)	DL Review	Multimodal DL approaches	Multiple	Varies	Broad review	Medium
^28^	Kikon & Bania (2024)	ML + Sentiment	Classifier w/sentiment scoring	Twitter	Good	Mixed feature signals	Medium
^20^	Zamani et al. (2023)	Rumor Detection DL	DL for rumor + fake classification	Twitter/News	Stable detection	Deployment complexity	Medium
GETE (Proposed Model)	Graph-Augmented Transformer Ensemble Framework	Robust and scalable fake news detection	Graph-integrated transformer ensemble	LIAR/FakeNews	High accuracy & scalability	Emerging threats, minor overhead	Minimal

Papageorgiou et al.^33^, for instance, investigated LLMs and deep neural networks for counterfeit news detection, highlighting the strength of transfer learning for semantic representation. While their approach focused primarily on text-based modeling, our framework extends this line of work by pairing semantic encoders with graph-based cues^8,13^, allowing joint exploitation of content and relational information. Beyond hybrid architectures, researchers have begun exploring innovative paradigms. Courtroom-FND simulates adversarial debate between roles (prosecution, defense, judge) to improve interpretability and veracity judgment^35^. Other work has investigated few-shot detection through adversarial and contrastive self-supervised learning^37^. The field has seen the development of new methods which include context-aware LLM prompting for veracity inference^34^ and multi-task prompting for consistency reasoning^38^. The methods require systematic thinking and flexible approaches which work together with ensemble-based frameworks such as GETE. Various studies on online information analysis research different aspects of this subject domain. These include figurative language detection in social media^39^, satire detection^40^, sarcasm detection^41^, hate speech^42^, and financial misinformation^43,44^. The research demonstrates particular problems in specific domains yet our system provides a universal framework which unites semantic and relational data for misinformation identification. Fake news detection has also progressed through multiple technical paradigms. Early approaches employed machine learning classifiers with handcrafted features^19,20^, later enhanced by word embeddings such as Word2Vec and GloVe^45^. With the success of deep learning, transformer architectures such as BERT and RoBERTa achieved state-of-the-art performance on benchmark datasets^23–25,46^. Sentence transformers^47^ and document embeddings^48^ further enriched representation learning for text-based misinformation detection.More recent works emphasize ensemble and mixture-of-expert (MoE) models, where specialized expert networks capture different aspects of the data and gating mechanisms dynamically combine their outputs^49^. Network-aware ensemble frameworks that incorporate propagation features have also been proposed^50,51^, showing that modeling how news spreads in social networks can enhance classification performance. Beyond detection, mitigation strategies have gained attention. Proactive immunization^52^, tree-based blocking^53^, and community-focused methods^54^ seek to reduce misinformation spread by targeting influential nodes or dense clusters. Real-time detection frameworks^55,56^ address the need for streaming analysis, combining scalable pipelines with fast inference. Additionally, virality prediction models^57^ aim to anticipate which content is likely to spread widely, enabling early interventions. Together, these diverse strands of work underline the growing sophistication of fake news detection research. Our contribution, GETE, is positioned within this landscape as a framework that adaptively integrates semantic and relational learning through a meta-learned ensemble mechanism, complementing prior approaches and addressing challenges of adaptability and robustness across domains.

### Problem formulation

Fake news refers to either fully fabricated narratives or partially manipulated statements designed to mislead, both of which require combined semantic and relational modeling to detect effectively. In the text content area, fictional news reports have subtle linguistic characteristics that make them different from real news reports. They may include sensationalized tone, emotional tone, or manipulative tone^24,32^. It is difficult to identify these characteristics as fake news stories may appear as real news from superficial appearance^33^. This is also compounded by the fact that the meaning of words and phrases is highly context-dependent. To mitigate this, transformer models like BERT^6^ and RoBERTa^33^ have fared well as they are able to learn the fine-grained dependencies as well as contextual word relations. These models lack the capacity to process individual articles in isolation of the rest, constraining their capacity to learn the global context of article dissemination within social networks as well as the source credibility^26^. Alternatively, GNNs provide another choice in that they represent the dissemination of news in a social network by users, articles, and sources as nodes and the interaction between them as edges^8,27^. GNNs are better suited to represent how the information is disseminated in the network according to what the users do and the authenticity of the sources^29^. They are lacking in representing the fine-grained linguistic semantics to figure out if the news content itself is deceptive^15,30^. One of the greatest challenges in identifying fake news, then, is how to effectively combine textual and relational features^12^. The conventional approaches that summarize only the latter may not necessarily be able to make their best use of their interactions^11^. An ensemble model that can leverage transformer-based models and GNNs^1,9^. The model is intended to be able to learn adaptively the relative ranking of textual and relational features based on the particular context of the news article and network it belongs to^13^. The solution of meta-learning provides flexible adaptation capabilities for handling environments that undergo changes. The model will learn to process different types of data and new fake news distribution patterns through its ability to combine text and relational signals^20,22,28^. Reducing the problem to learning to create a classifier with maximum accuracy by fusing the features of the two modalities in the appropriate manner, learning to update the transformer and GNN model weights according to the task, makes the detection system not only accurate but also robust and adaptive to changing misinformation tactics^7^.

### Current status and limitations of existing methods

Existing approaches to fake news detection can be broadly categorized into three groups: (i) traditional machine learning models (e.g., SVM, Logistic Regression) that rely on handcrafted linguistic and statistical features^19,20^, (ii) transformer-based models such as BERT and RoBERTa that excel at semantic comprehension but process articles in isolation^23–25^, and (iii) graph-based models (e.g., GCN, GAT) that model propagation structures and social interactions but often underutilize textual semantics^4,8,15^. While these methods have advanced the field, they face notable limitations, including poor cross-domain generalization, vulnerability to data sparsity, and lack of adaptability across different misinformation contexts^21,28,33^. The proposed GETE framework addresses these limitations by introducing a meta-learned ensemble strategy that dynamically integrates semantic representations from transformers with relational dependencies captured by GNNs. Unlike prior static ensembles, GETE adaptively adjusts its reliance on each modality based on validation feedback, thereby improving robustness, scalability, and cross-domain applicability.

## Proposed framework

**Fig. 2:**
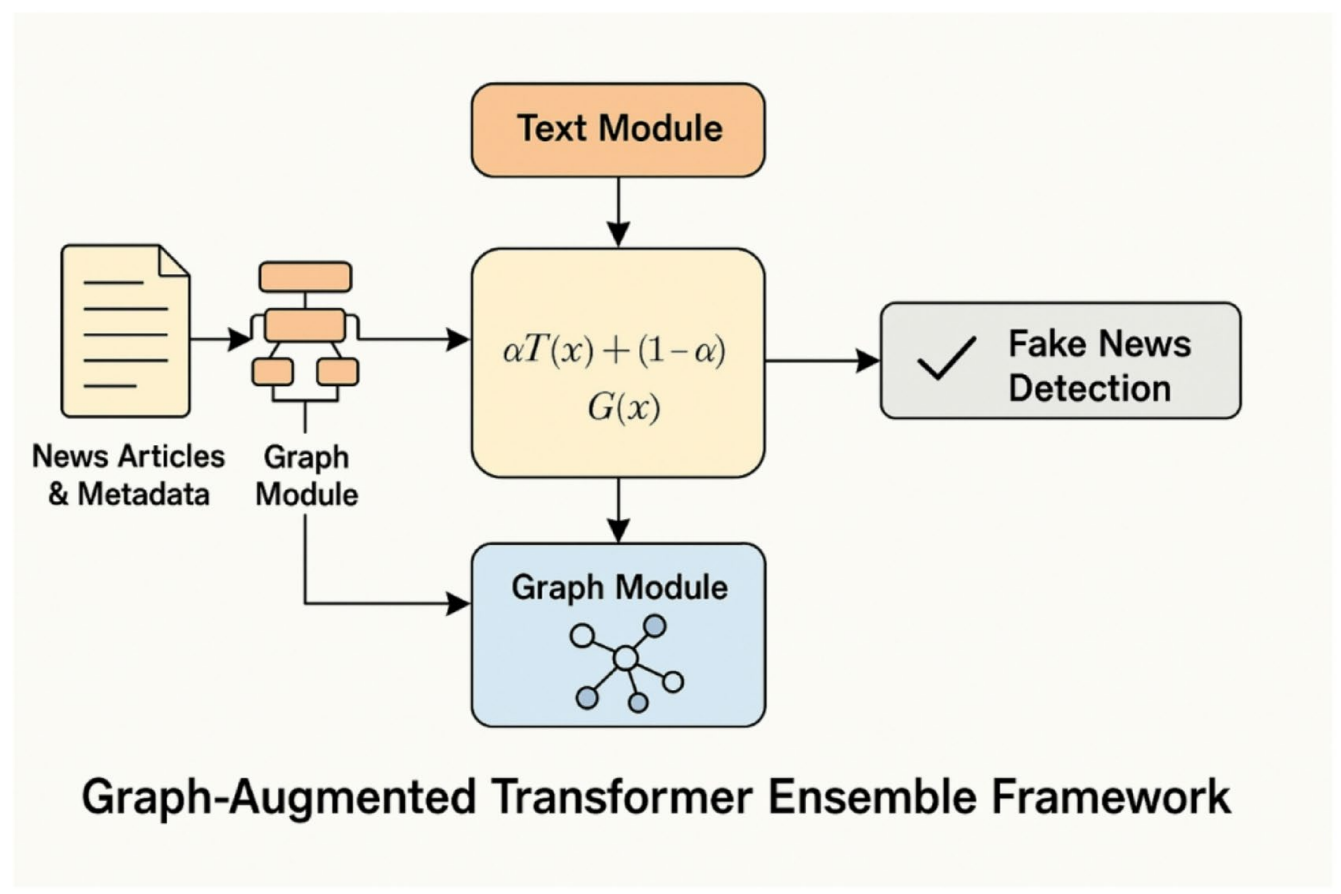
Graph-Augmented Transformer Ensemble framework.

This section presented our hybrid fake news detecting system that combines transformer technology for semantic analysis with graph learning, connected by an adaptive process. This adaptive learning process is referred to as a meta-learned ensemble and has been explored in^1^ and^31^. This suggested method is designed to exploit the best features of each approach. Specifically, it combines two distinct techniques, deep semantic analysis provided by transformer models and relational reasoning enabled by graph neural networks (GNNs). The overall framework architecture consists of three core components: a transformer, a GNN, and an additional GNN module. We begin by employing a transformer (BERT or RoBERTa) model. They are responsible for extracting semantic features from the articles’ textual content^6,23^. The transformer models are especially good at learning how elements of a text interrelate with each other, and aiding the revelation of meaning behind each word, each sentence, each paragraph^10^. This capability is especially useful for detecting the nuanced wording and deception often used by misinformation^32^. To complement this, we also employ a GNN which captures the relationship between the users, the articles and the sources, following the precedent set by prior research^8,14^. In the modern online era, how fake news propagates frequently relies upon user actions and the character of the sources which are propagated^4,27^. Organizing this data as a graph, with the users and articles as the nodes, and the interactions between them as the edges, the GNN may learn trends in the spread of information and the level of user interaction with it^15^. This matters since false news often propagates as a coherent unit over a set of networks, and this sort of dynamic understanding holds the key to successful detection^28,30^. But whereas GNNs excel at modeling relations, they don’t completely encode the deeper meaning of language, this is rectified by transformer models, which come into play and cover the gap^29^. The system also makes use of the meta-learned ensemble module as the third constituent. The module self-adjusts the weight of each model’s output, depending on how the models perform on the validation set^9,31^. Instead of just blending the models with fixed weight, as is standard for traditional ensembles, the meta-learned ensemble makes use of a unique technique for picking the optimum mix of the models^3^. Owing to the dynamic weight, the system can cope better with data change and also get tougher^22^. For instance, when textual features significantly suggest fake news, the ensemble places more emphasis on the transformer model; when relational cues from user interaction prove more informative, the GNN constituent gets the focus^11^. This adaptability makes the ensemble generalize into different elements of fake news in other applications^20^. The combination of the three elements, transformer-based textual analysis, graph-based relational learning, and weighting of the ensemble aided by metalinguistic knowledge forms a successful and transferrable framework used for determining fabricated material^1,12^. Through the use of the content as well as the context, the approach may distinguish deception from non-deception material with increased efficiency compared with methods directed at each specific class of signals singly^21,24^. Further, the meta-learning aspect of our ensemble allows for the possibility of continued fine-tuning and improvement, and the system may thus appear as a part of real global applications wherein false trends of news continuously change and evolve^7,25^. The architecture starts with preprocessing where text as well as features based on graphs are extracted from the input data^33^. Figure 2 depicts the overall architecture of the Graph-Augmented Transformer Ensemble (GETE) framework. The textual data is pre-processed using a standard pipeline^23^. The preprocessing steps include lowercasing, removal of punctuation and special characters, elimination of stopwords, tokenization, and lemmatization. Additionally, URLs, mentions, and hashtags are normalized to maintain consistency. This preprocessing ensures that the textual input is clean, semantically meaningful, and ready for feature extraction using word, sentence, or document embeddings.

### Transformer-Based text embedding

The suggested framework combines the semantic strengths of transformer models with the relational insights of GNN, all integrated through an adaptive, meta-learned ensemble. It consists of three core components: A transformer model (BERT or RoBERTa) that extracts contextual features from the text^2,23^. A Graph Neural Network (GCN or GAT) that models the structural relationships within user-article-source interaction graphs. A meta-learned ensemble layer that dynamically adjusts the weighting of each model’s output to optimize overall performance through validation-based learning. Transformers use self-attention to learn long-range word dependencies^6^. For a tokenized input sequence x = {x_1_,x_2_,.,x_n_}, each token is transformed into a contextual embedding: hi = Transformer(xi) for i∈[1,n]^23^.

### Graph neural network module

The GNN module we propose helps the model understand how users, news articles and sources are related to each other. By being shared, liked, commented on and linked to by users, this type of news can spread very quickly. Just looking at the text is not enough, as these interactions involve the connections between many actors. In our framework, the heterogeneous graph is formally defined as G = (V, E), where nodes V represent entities {users, news articles, sources}, and edges E capture different types of relationships. Specifically, user nodes encode behavioral features such as posting or sharing histories, article nodes contain semantic embeddings extracted from the Transformer model, and source nodes encode metadata such as domain credibility scores^4,8^. Edges are created by (i) user–article interactions (e.g., sharing, liking, commenting), (ii) user–source subscriptions or follows, and (iii) article–source references (e.g., citations or publishing links)^2,28^. To assign meaningful weights to user–article interaction edges, we combined engagement indicators using a weighted scoring scheme. Specifically, the edge weight was computed as: 0.60 × normalized shares + 0.30 × normalized comments + 0.10 × normalized likes. Shares were given the highest importance because they typically indicate stronger endorsement and contribute more directly to misinformation spread. All engagement features were min–max normalized to the range [0,1] before combining them, and the final weight was again scaled to [0,1] to maintain uniformity across interaction types. Relationship extraction is automated from the raw dataset logs. User activity fields (shares, likes, comments) define user–article links, while metadata tables define article–source mappings. Source credibility scores are derived from third-party credibility databases or annotated subsets, ensuring that the graph captures both behavioural and credibility-based relationships^21,26^. Processing such graphs at social media scale introduces challenges such as high sparsity, skewed degree distributions, and memory bottlenecks. To mitigate these, we employ mini-batch training with neighbour sampling (GraphSAGE-style) and sparse adjacency storage^14,15^. Additionally, edge-type normalization ensures fair contribution across heterogeneous relations, while GPU-based batching enables scalability across millions of nodes^6,7^.

They are particularly advantageous at learning how different entities are connected which is shown graphically with nodes for each item and edges for the relationships (e.g., users sharing an article, following a user or citing a source)^4,29^. Our model views the fake news detection problem as a heterogeneous graph, with data nodes represented by V and data edges represented by E. Users, articles and sources are some of the most common entities in the graph of Facebook. The edges stand for anything users or articles do, for example, commenting on an article, sharing a source, following a source platform or linking an article to a different source^28^. Every node v∈V contains a feature vector xv​ that collects the characteristics and properties of its related entity. A typical user node could hold their recent activity details, but an article node might contain information in the textual format provided by the transformer model^11^. Due to the aggregation process, the GNN propagates updates from neighbouring nodes to refine node properties. The model retains both node features as well as the inherent interactions between the nodes, which matters for detecting the diffusion of misinformation through networks^25^. In the workflow described by us, this aggregation uses either Graph Convolutional Networks (GCNs) or Graph Attention Networks (GATs). GCNs effectively learn structural features of local neighbourhoods^1^. In some scenarios, though, GATs employ an attention model which pays more importance to particular connections, like those between influential users, compared to others^32^. Updated node embeddings provide a useful summary of entities in the graph, showing what a node is and how it interacts with others. Nodes that often share information from a reputable source are more likely to be trusted and considered important in the network, as discovered in this study^7,20^. An article that many reliable sources have shared and referred to may be judged more trustworthy by parliamentary libraries^13^. Seeing things with graphs is very useful for learning the ways in which misinformation is shared. A lot of the time, fake news is spread further by some users, sources or groups and this spread is caught by the GNN model using amplification patterns^27^. Articles that have been shared by people with a record of misleading information or by sources with a poor track record may be designated as fake news. Using input from different areas of a network, GNNs have been shown to excel at finding such propagation patterns, something transformers might have a difficult time spotting^31^. The GNN module functions together with the transformer-based text embedding module in parallel. While the transformer pays attention to the meaning of the articles, the GNN studies how the network’s components are related^12,21^. Semantic analysis and relational modeling are merged in the ensemble module, with the former adding extra weight to the approach that performs best^1,9^. As a result, the GNN module improves the framework’s identification of fake news by studying the relationships between users, articles and sources^2,26^. The DeepMoji model can generate new insights by using relationships among data which, once integrated with the deep text learning from transformers, makes the fake news detection system work more accurately and steadily^22^. Equation (1) expresses the core message-passing mechanism of a GNN layer. Its primary purpose is to define how a node in the graph updates its representation (feature vector) based on information from its neighbours.

1 $$\:{h}_{v}^{\left(l+1\right)}=\sigma\:\left({\sum\:}_{u\in\:N\left(v\right)}\frac{1}{{c}_{vu}}{W}^{\left(l\right)}{h}_{u}^{\left(l\right)}\right)$$

Where $\:{h}_{v}^{\left(l+1\right)}$denotes the feature vector of node v at layer l + 1, while $\:{h}_{u}^{\left(l\right)}$​ represents the feature vector of a neighbouring node u at layer l. The set N(v) includes all neighbours of node v in the graph. The weight $\:{W}^{\left(l\right)}$ is a learnable parameter that transforms the features from the current layer. The term c_vu_ is a normalization constant, often computed based on the degrees of the nodes v and u, which ensures that feature aggregation remains stable and unbiased across nodes with varying degrees. The non-linear activation function σ(⋅), often ReLU, introduces non-linearity to the model. The encoding allows the GNN to iteratively aggregate and transform neighbour node information, thus preserving local structure as well as context of features.

In our framework, the heterogeneous user–news–source graph is constructed with three distinct types of edges that capture various interaction dynamics within the social media ecosystem. The User–Article edges are established when a user interacts with an article, such as by sharing, liking, or commenting on it, reflecting direct behavioral engagement. The User–Source edges represent a user’s subscription or follow relationship with a particular news outlet, capturing long-term trust or interest patterns. Finally, the Article–Source edges connect news articles to their respective publishing domains or cited sources, thereby encoding the credibility and provenance of the information.

Each edge is assigned a weight that reflects the strength and importance of the relationship. For User–Article links, weights are computed based on the frequency and intensity of interactions, while for User–Source and Article–Source links, the weights are derived from source credibility scores obtained from annotated or external databases. These raw weights are further normalized into the range [0,1] to ensure a balanced influence among heterogeneous edge types and to prevent any single relation from dominating the learning process.To mitigate the impact of graph sparsity—a common issue arising from incomplete or uneven user engagement—we employ mini-batch neighbor sampling inspired by the GraphSAGE framework, along with sparse adjacency matrix storage for computational efficiency. Moreover, edge-type normalization is applied to balance the contribution of dense and sparse relations, preventing the model from overemphasizing frequently occurring node types. This heterogeneous graph structure enables the GNN to effectively learn relational dependencies among users, articles, and sources while maintaining balanced representation even under sparse social interactions.

### Meta-Learned ensemble module

Rather than relying on fixed weights or simple averaging of the outputs from the Transformer and GNN models, our approach employs a meta-learning strategy^22^ to dynamically learn the optimal combination of these outputs for improved robustness and adaptability. Let the result^1,12^ from the Transformer model be defined as pT = Softmax(Linear(Tx​)) and from the GNN be defined as pG = Softmax(Linear (Gx​)). For the result of the final prediction, we use y^=α⋅pT+(1 − α)⋅pG​, where α is a learnable parameter in range [0, 1]. This is optimised with validation to enable the ensemble to adaptively vary its dependency on each model’s result in order to enhance robustness and flexibility^20^. These probability distributions are combined to form the final prediction is made, but not statically or rigidly. Rather, a meta-learned weight α determines how much weight each of the forecast should have^31^. The overall prediction is generated with a combination of the outputs of the Transformer and GNN models, denoted as pT​ and pG respectively​. The weight α for this combination is learned using meta-learning. This weight reflects the relative confidence of the Transformer and GNN models in their predictions^9,11^. A validation set is used to optimize α, with the goal of minimizing the loss function and thereby improving the ensemble’s performance^3^. The output of the Transformer model is defined as T(x), a transformed feature vector extracted from the input text, with the predicted class probabilities represented by pT​. Similarly, the output of the GNN model is represented asG(x), a feature vector capturing the relationships among various nodes (e.g., users, articles, and sources) in the social graph^4,8^. Equation (2) illustrates how the meta-learned ensemble combines these outputs:

2 $${y^ \wedge }=\alpha *pT+(1 - \alpha )*pG$$

In the proposed GETE framework, the ensemble weight α is implemented as a learnable model parameter and is optimized end-to-end along with the Transformer and GNN components. Rather than relying on a separate external validation loop, α is updated directly through backpropagation during training. It is initialized within the interval [0,1] and its gradients are computed from the same cross-entropy loss used for optimizing the remaining model parameters. This mechanism enables α to dynamically adjust the relative contribution of semantic features (from the Transformer) and relational features (from the GNN), based on their effectiveness during training. To ensure generalization and avoid overfitting, we monitor α using validation performance through early-stopping and learning-rate scheduling. This provides a stable and reproducible optimization process.

Unlike prior hybrid architectures such as GETAE^21,33^, which employ fixed or heuristically tuned ensemble weights, the proposed GETE framework introduces a dynamically learned ensemble mechanism. The ensemble weight α functions as a trainable parameter which backpropagation optimizes end-to-end to determine the optimal balance between Transformer-based semantic features and GNN-based relational features during training. The dynamic weighting strategy produces better results for robustness and generalization across different domains. GETE achieves both real-world scalability and avoids static fusion limitations through its end-to-end training approach which uses validation feedback^11,28,31^. The ensemble weight α is optimized jointly with the Transformer and GNN parameters during end-to-end training. Specifically, the final prediction y^=α⋅pT+(1 − α)⋅pG is compared against the ground-truth labels using the standard cross-entropy loss. Gradients with respect to α are computed via backpropagation, ensuring that α dynamically adjusts to balance semantic (Transformer) and relational (GNN) contributions. Unlike meta-learning frameworks such as MAML or Reptile, which require inner/outer optimization loops across multiple tasks, our approach directly treats α as a trainable parameter within the classification objective. Conceptually, this resembles a stacked ensemble or mixture-of-experts gating mechanism^1,6,22^, where α functions as an adaptive weight that minimizes classification error.

Here, y^​ shows the equation’s prediction which comes from adding transformed versions of both the transformer’s output probability distribution and the GNN’s output probability distribution. Each model receives a dynamic weight of α ∈ [0,1] which is trained to influence the contribution from the ensemble^22^. In this case, α is a tweakable parameter between 0 and 1, obtained as a result of validation optimization. To be exact, pT is the distribution made by the transformer model, pG is the distribution generated by the GNN and α controls how strongly the models’ results are blended. Equation (3) outlines how the objective for optimization works when finding the right weight α on validation data:

3 $$\alpha \leftarrow {\mathrm{argminL}}(\alpha \cdot {\mathrm{pT}}+\left( {{\mathrm{1}} - \alpha } \right) \cdot {\mathrm{pG}},{\text{ }}{{\mathrm{y}}_{{\mathrm{val}}}})~~~~~~~~~~~~~~~~~~~~~~~~~$$

In Eq. (3), L(⋅) denotes the loss function, typically cross-entropy, which measures the discrepancy between the combined prediction α⋅pT+(1 − α)⋅pG and the true labels yval from the validation set. The optimization seeks the value of α\alphaα that minimizes this loss, thereby learning the most effective way to weigh the transformer and GNN predictions for improved generalization and accuracy. Equation (4) expresses the final prediction after the ensemble weight α has been optimized:

4 $${{\mathrm{y}}_{{\mathrm{pred}}}}={\text{ }}\alpha \cdot {\mathrm{pT}}+\left( {{\mathrm{1}} - \alpha } \right) \cdot {\mathrm{pG}}$$

For reproducibility, we emphasize that α is trained as part of the network parameters during end-to-end learning, and no nested meta-optimization loop is required. The optimization follows standard gradient descent, ensuring a transparent and easily implementable training process. It is important to note that while hybrid Transformer–GNN frameworks such as GETAE^31^ have been previously proposed, they typically employ static or validation-based weighting strategies. In contrast, GETE treats the ensemble weight α as a trainable parameter, optimized end-to-end with the classification objective. This dynamic optimization allows α to adapt continuously during training, making GETE more flexible and resilient than earlier approaches^21,33^. Unlike fixed fusion strategies used by GETAE^31^, which apply static or pre-defined weights to combine transformer and graph embeddings, GETE treats the ensemble weight α as a trainable parameter that is optimized jointly with all model components via backpropagation. The model achieves performance stability across different datasets and adapts to changing fake news patterns through its dynamic weighting system which adjusts the influence between semantic and relational modules. Here, ypred is the ensemble’s result that is used to make predictions. It reuses the optimized weight α∗ previously obtained from Eq. (3). It is both the relational dependencies learned by the GNN and the semantic features learned by the transformer, leading to powerful and robust prediction. The meta-learned ensemble module offers several key advantages. The model achieves optimal performance by dynamically adjusting the weight of each sub-model prediction to match different datasets and scenarios which results in improved robustness and accuracy^6,26^. The model achieves effective generalization across different types of misinformation because it can modify its use of transformer-based or graph-based methods according to validation feedback^21,25^. By leveraging the complementary strengths of transformers and GNNs, the ensemble model achieves higher predictive performance than models based on a single modality^2,29^. The model adjusts to fake news evolution by determining the best weight distribution for each ensemble component. The method combines the advantages of both architectures through their unique capabilities which address the limitations of each other. The learned ensemble weights enable the model to handle different and new challenges in fake news detection according to^13^ and^15^.

The end-to-end pipeline of Algorithm 1 uses natural language processing and graph learning and ensemble modeling to detect fake news according to best practices. The first step involves collecting raw news feeds which need preprocessing to standardize text content and divide them into separate tokens while removing unnecessary symbols and noise^10,19^. After cleaning, the input is fed through strong feature extractors such as transformer-based language models (e.g., BERT, RoBERTa) that extract strong contextual semantic long-range dependencies^23,31^. The social network activity patterns and information spread dynamics of users are modeled through GNNs which require both relational network structures and diffusion mechanisms to detect disinformation. For strong decision making, the resultant of these diverse feature representations is combined through inter-modal or multi-modal approaches blending textual, visual, and relational signals^6,24^. The model becomes able to detect fake content characteristics that individual modalities cannot detect on their own through this combination. Then, a meta-learning ensemble mechanism pools the predictions from several base learners like CNNs, LSTMs, GNNs, and transformers^1,12,22^. The model applies this layer to change output weights during execution based on confidence levels and contextual reliability for enhanced performance on new data. The system produces two classification results for real and fake content with their respective confidence levels. The system design contains independent modules which enable adaptable operation with various datasets for deployment across various platforms and media types. In practice, α is optimized through gradient descent as part of the model’s learnable parameters, and no separate outer optimization loop is required. Validation metrics are used only for monitoring and early-stopping purposes, not for explicit gradient-based optimization.

**Algorithm 1:**
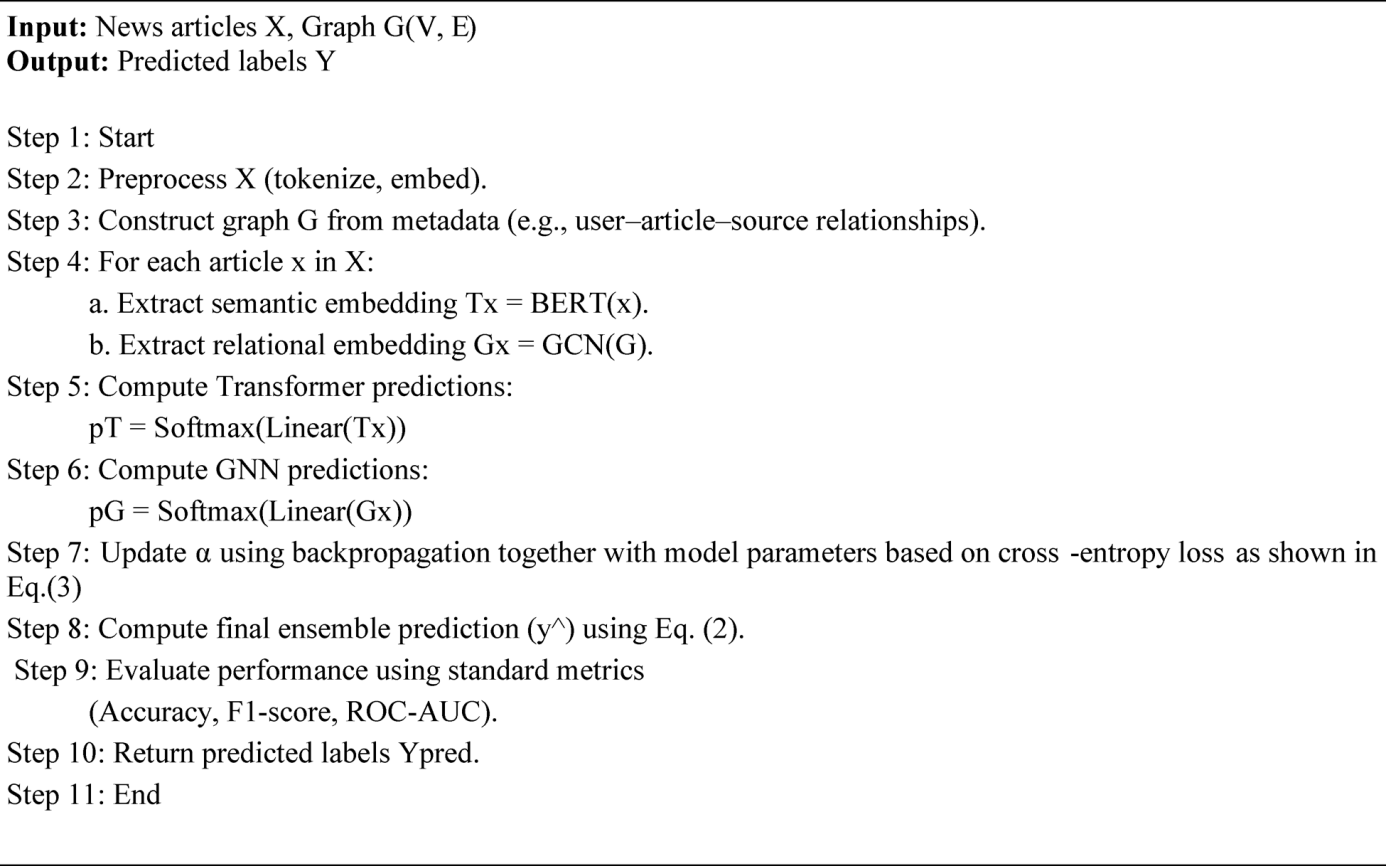
Graph-Enhanced Transformer Ensemble (GETE) for Fake News Detection.

### Final architecture

**Fig. 3:**
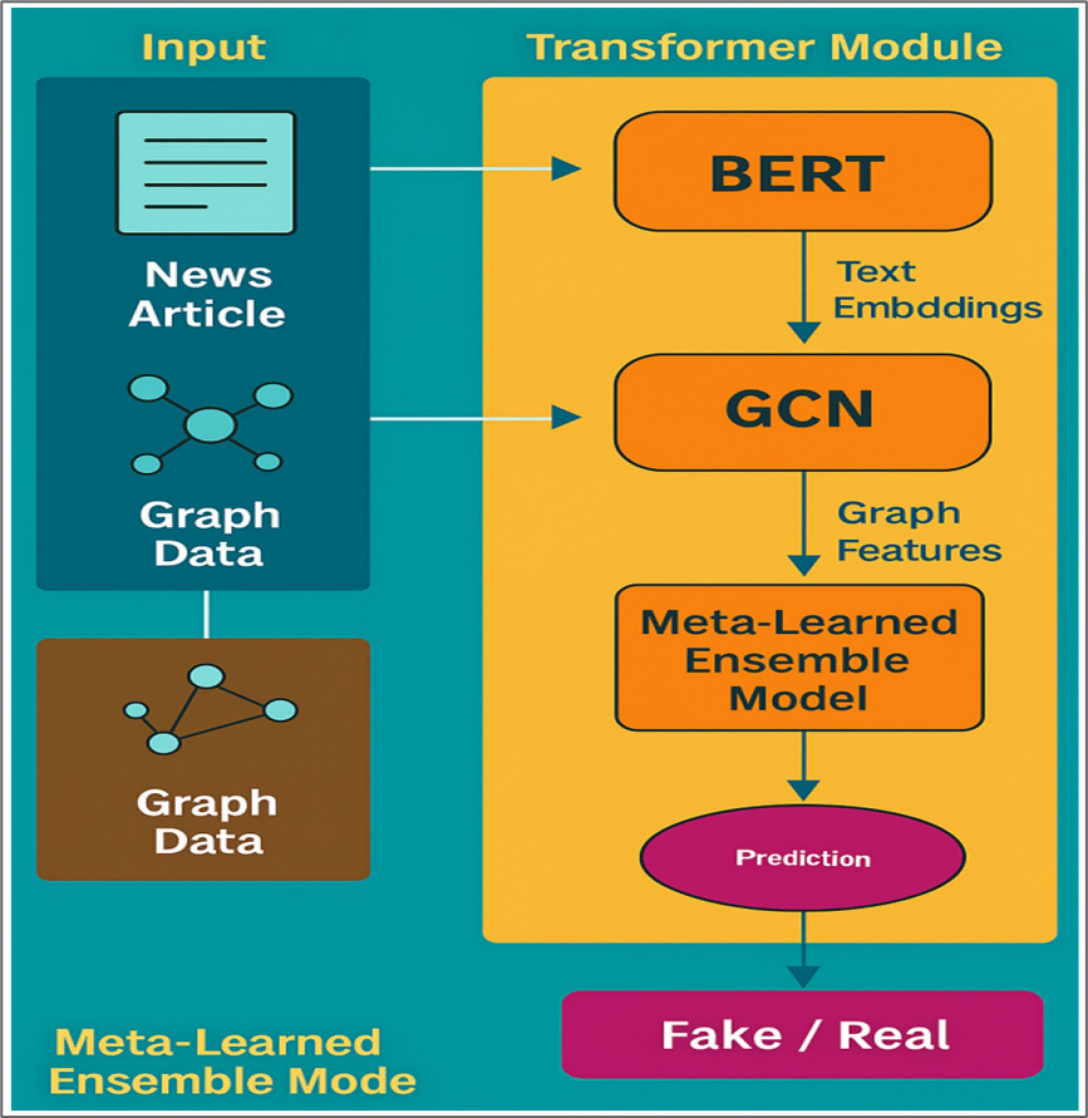
GETE for Fake News Detection follows the architecture.

the final architecture of framework combines the transformer, the GNN and the meta-learned ensemble into a system that can efficiently search and identify fake news. Due to the transformers and the GNNs, the system can model the text in messages and the rumours spreading in social networks^2,8^. The system operates on news articles and the underlying graph that indicates how users, articles and sources are related to each other in the first phase. The preprocessing phase determines useful features from the text content and from the graph networks. The layer then operates on the text into a form that serves the purpose of the transformer model and the graph is structured in a way as to store any interactions and connections to serve the purpose of identifying fake news. Then the structure performs two workflows simultaneously: one for the transformer model and another for the graph neural network. Most of the applications of this methodology rely on the transformer model to enable the system to derive deep meanings from the content of the news article^23^. The self-attention of the transformer allows it to view the deep relationships between various words in the text^10^. The disadvantage is that the GNN module discovers how the users, articles, and sources are connected to one another within the network by using the graph data^29^. Being a graph model, the GNN is able to view how misinformation is spreading and identify any patterns that seem to exist because of social media connectivity^4,13^. Both models, once trained, generate outputs which are input to the meta-learned ensemble module. The module adapts itself as new data gets published. scales each model’s output by a learned weight, α. meta-learning is applied on the validation set in order to discover it^3,9^. If the ensemble weight is learned optimally, the method effectively combines the most informative features from both models while minimizing the overall prediction error. This ensures that the final prediction, derived from the weighted combination of outputs, is as accurate and reliable as possible^11,20^. The output of the ensemble module is the predicted label, either real or fake. The system makes use of the right and standard evaluation metrics like accuracy and the F1-score. ROC-AUC clearly shows the capability of our system to identify fake news against other approaches. using systems^12^, and^7^. Figure 3 shows the workflow of the GETE system for fake news detection, integrating text, graph, and ensemble modules. The integration layer optimizes α jointly with all network components, allowing the model to balance semantic and relational cues without requiring any external optimization loop.

### Datasets

We tested our proposed hybrid method using two broadly recognized datasets (LIAR, FakeNewsNet) that are commonly used in fake news detection research^10,24^. These datasets allowed us to assess both the semantic content of the text and the relational patterns in how fake news spreads^13,27^. Below, we provide a more detailed explanation of the two datasets used.

i) **LIAR Dataset**: LIAR dataset is a common dataset used in fake news detection and consists of 12,836 short political statements taken from fact-checking websites like PolitiFact^15^. The statements are described as in one of six truth levels, ranging from “false” to “pants-fire,” “barely-true,” “mostly-true,” and “true”^26^. This definition aligns with the LIAR dataset’s labeling scheme, where fabricated and highly inaccurate statements fall under “False,” while partially true or misleading statements fall under intermediate labels. The metadata also contains information regarding the speaker, the subject matter of the statement, as well as the context in which the statement is being made. Such metadata can be helpful in determining how effective it is in terms of context as well as topic-based disinformation^22,30^. For instance, accusations concerning various public figures or parties can activate some linguistic tendencies or biases that can impact the model’s performance^14^. The information enables us to examine the extent to which the method handles sentences that have no evidently perceivable symptoms of falsehood but need greater linguistic and situational awareness^23,29^. We transform the dataset into a binary classification task in a manner such that it approaches real-world situations as closely as possible, where disinformation will be labeled as neat “True” or “False” containers^33^. The simplification categorizes the groups “mostly-true,” “half-true,” and “true” in “True,” and barely pants-fires, false and true under “False”^5^. LIAR dataset is a good test for measuring how well the model can detect fine misinformation in short messages which have been characteristic of political disinformation. It is especially good at evaluating transformer models like BERT and RoBERTa for text understanding capacity^4^.
ii) **FakeNewsNet Dataset**: FakeNewsNet dataset is one of the biggest and most comprehensive datasets capable of identifying fabricated news^4,8,27^. It supports both content-based and relational (graph) data. Thus, making it appropriate for measuring hybrid models containing text and network variables^6,13,30^. FakeNewsNet integrates news articles from two reputable fact-checking sites, PolitiFact and GossipCop, and a set of metadata, including publisher name, user interaction (likes, retweets, etc.), and social propagation measures^15,26^. This social interaction information is accumulated in the form of graphs, with nodes as sources, articles, and users, and edges as relationships between them entities^14,29^. The most significant advantage of the FakeNewsNet dataset is that it contains propagation graphs, which are representations the sharing of articles through social networks^31^. This functionality allows us to study the graph-based aspect of our hybrid method, i.e., the ability of Graph Neural Networks (GNNs) to learn the social dynamics of false news dissemination^22^. The temporal features are also present in the dataset, i.e. we can see how the dynamics of spreading fake news evolve over time^20^. This is particularly key to the identification not just of false news, but of developing or adapting misinformation that circulates between different user groups^28^. FakeNewsNet is helpful in ascertaining how much the proposed model makes use of text information (from the articles) with relational signals (of user interaction and source trustworthiness) in an evolving social environment^21,23,30,31^. It also provides a varied collection of articles of varying levels of reliability, which helps in measuring the extent to which the model generalizes to other news content^19^.

The two datasets present different testing scenarios which help us evaluate the effectiveness of our proposed model. The LIAR dataset is particularly concerned with text-based disinformation within the framework of a controlled, political context^2,23^, while FakeNewsNet offers a realistic environment to learn how news stories propagate and change over time with social media networks^7,8,29^. The model receives testing from both text-based and relationship-based evaluations which enables us to measure its ability to detect false news effectively^1,3,9^. They also form a good basis for comparing different fake news detection methodologies. Although LIAR emphasizes the work of textual semantics and political bias^10,24^, FakeNewsNet emphasizes the role of social dynamics and user actions in the diffusion of misinformation^4,22,31^. Table 2 summarizes the LIAR and FakeNewsNet datasets including article count, average length, and graph details.

**Table 2:** Dataset Summary.

Dataset	Articles	Avg. Length	Graph Edges	Source
LIAR	12,836	17 tokens	N/A	Politifact
FakeNewsNet	22,000+	280 tokens	1.3 M+	Twitter API

It is important to note that in this work we restrict the LIAR dataset to a binary classification setup (‘True’ vs. ‘False’), following prior studies such as Wang (2017) and Bhatt et al. (2021). The original six-class labels were merged into two groups to better reflect real-world misinformation detection settings. Experiments on the full multiclass setup are beyond the scope of this paper.

### Preprocessing

 Preprocessing is required before feeding the raw data to the model and involves a series of transformations actions that are aimed at processing text and graph data^30^. At the text processing phase, the text data is tokenized using the BERT tokenizer^2,6^. The tokenizer divides the text into tokens and transforms it into a form that is compatible with the BERT model. Since BERT models are case-sensitive, the uncased version of BERT is employed, i.e., all the characters are lower case^10,23^. In a try to have fixed input size throughout different documents, a token length constraint of 256 is utilized^24,32^. After tokenization, non-ASCII characters are removed to avoid any interference in model processing so that the text is readable and understandable. Also, stopwords that are common, and are normally not required for understanding the core meaning of a sentence, are removed using the Natural Language Toolkit (NLTK)^33^. For stop-word removal, we used the standard NLTK English stop-word list, supplemented with an additional set of platform-specific tokens such as “rt”, “via”, and common URL fragments (e.g., “http”, “https”, “www”). This combined stop-word list helps remove non-semantic artifacts common in social-media text. The maximum token length for each article was set to 256 tokens, and the BERT uncased tokenizer was employed for consistent text normalization. This assists in reducing noise within the data in a way that the model can concentrate on more significant terms^7^. Simple text cleaning, such as removing special characters and unwanted punctuation, is also done to enhance the quality of the input data^20,25^. In the construction step, a graph is built to illustrate the relationships between various entities, as model uses GNNs^13^. The graph has three types of nodes: users, articles, and sources. They are connected with edges that represent interactions, i.e., user-shares-article or source publishes-article^15^. The figure is plotted using the NetworkX library, a Python library that is meant for construction and manipulation of complex networks. PyTorch Geometric is also used for processing the graph data in deep learning such that the model can handle graph-based data structures^27,29^. Extracting features is the most important aspect of enhancing the ability of the model to distinguish between genuine and artificial information^4,22^. User-level characteristics consider the age of the account, as this is utilized in the determination of the credibility of a user based on how long they have been active on the site. The user’s number of followers is another important feature, providing an indication of their dissemination and influence in the network^19,31^. Tweeting activity, which tracking the frequency at which a user tweets or interacts with material, is also present, as it may indicate a user’s frequency of influence or credibility^15,28^. For articles, various features are extracted that aid in the assessment of their trustworthiness. Text length is one of them features, because longer, more detailed articles will be read as more authoritative than short, sensationalized ones^9,11^. Subjectivity of the articles is also assessed, since extremely subjective language may be evidence of fraudulent or biased content^3,26^. Publisher bias is also an important feature, since the political orientation of the publisher may affect the validity of the article^5,20^. In treating the data in this manner, both textual and relational features are accurately captured, such that the hybrid model can effectively identify false news^1,12^. Table 3 provides an overview of different fake news detection approaches highlighting their strengths, limitations, use cases, complexity, interpretability, scalability, and performance. The datasets became more understandable through our exploratory data analysis (EDA) which included topic modeling and community detection and contextual cue analysis. We tested different weighting techniques for topic modeling^30^ to identify the most suitable method for extracting vital topics. The researchers used community detection methods^9^ to group documents into clusters which exposed hidden patterns in the social network structure. The researchers used entity mentions and hashtags and temporal patterns from the context to enhance topic representation which led to improved results^4^. The EDA provides researchers with knowledge about document topic distribution patterns and document relationships which guides the creation of models and evaluation techniques.

**Table 3:** Summary of models and their descriptions used in fake news Detection.

Approach	Strengths	Limitations	Use Cases	Complexity	Interpretability	Scalability	Performance
**Naive Bayes**,** SVM**	Fast, interpretable, simple to implement	Limited semantics, poor generalization	Basic classification, small-scale datasets	Low	High	Low	Moderate
**CNN/RNN**	Captures patterns and sequences, handles text effectively	Limited context range, high overfitting risk	Sequence modeling, text classification	Medium	Moderate	Moderate	High
**BERT/RoBERTa**	Deep semantic understanding, context-aware	Ignores network context, computationally intensive	Complex text understanding, sentence-level tasks	High	Low	High	Very High
**GNNs (GCN**,** GAT)**	Models’ relationships, social propagation, network-level insights	Weak on textual semantics	Social network analysis, fake news propagation	High	Low	High	High
**Existing Ensemble Models**	Improved performance using hybrid features	Often non-adaptive and dataset-specific	Multi-modal detection, hybrid systems	Medium to High	Low to Moderate	Medium to High	Very High

### Exploratory data analysis (EDA)

To better understand the characteristics of the LIAR and FakeNewsNet datasets, we conducted an exploratory data analysis (EDA). Table 4 presents descriptive statistics, including the number of documents per class, average and maximum document length, and vocabulary size. LIAR consists of 12,836 short political statements, averaging only 21 words per statement, which highlights its suitability for testing transformer-based semantic models. FakeNewsNet, by contrast, contains over 22,000 articles with an average of 412 words, offering a much richer semantic space. Moreover, FakeNewsNet includes social graphs with over 1.3 M edges, which is crucial for evaluating the relational reasoning capacity of GNNs. Figure 5 shows the class distributions for both datasets. LIAR is relatively balanced between True and False labels, while FakeNewsNet exhibits a slight skew toward Fake. Length distribution histograms further show that LIAR claims are short and concentrated, while FakeNewsNet articles vary widely in length, reflecting differences between claim-level and article-level datasets.We also analyzed thedistribution of unigrams and bigrams. In LIAR, the most frequent unigrams included political entities (e.g., “president,” “congress”), while FakeNewsNet frequently contained sensational bigrams such as “breaking news” and “shocking claims.” These linguistic signals, combined with relational propagation patterns, motivated the design of our hybrid approach.Overall, EDA highlights that LIAR challenges models with very short, context-dependent statements, while FakeNewsNet demands integration of both textual semantics and social graph structures. These insights informed the architecture of our proposed GETE framework.

**Table 4:** Ablation study on FakeNewsNet dataset.

Configuration	Accuracy (%)	F1-score (%)	ROC-AUC (%)
Transformer Only (BERT)	91.8	91.5	92.8
GNN Only	90.1	89.7	91.0
Transformer + GNN (no ensemble)	94.0	93.7	95.0
Ensemble (no meta-learning)	95.2	95.0	96.1
Full GETE (with meta-learning)	96.3	96.1	97.1

### Evaluation metrics

The performance of proposed model was evaluated using standard classification metrics, offering a comprehensive view of how well it performs across various domains. Accuracy (Acc) is the ratio of correct predictions, illustrating how well the model performs in general^3,32^. Precision (P) measures the ratio of correctly classified real news among all predictions made as real news. This is important when the cost of false positives is high, especially in areas like politics or public health^6,9^. Recall (R) estimates the fraction of actual real news that the model picks up correctly, and this is significant in the instance where missing actual news (false negatives) is detrimental^15,23^. The F1-Score (F1) is the harmonic mean of precision and recall, providing a balanced estimate in the situation of unbalanced classes because it takes into account both false negatives and false positives^1,21,22^. In addition to these fundamental measures, ROC-AUC is used to measure the discriminatory power of the model to classify between the classes of true and fake news^14,25,31^. It plots the true positive rate (recall) against the false positive rate, and the area under the curve (AUC) is a general indicator of the discrimination ability of the model^12,30^. In order to further analyse the performance of the model, confusion matrices are used to show the predicted against actual classes, helping to identify where the model may be failing. Precision-recall curves give more information on how the model sacrifices precision and recall at various thresholds, particularly for imbalanced datasets where accuracy can be not suitable to apply 11]. Finally, considering misclassified examples is helpful for determining the circumstances in which the model fails and for guiding future improvements^11^.

**Fig. 4:**
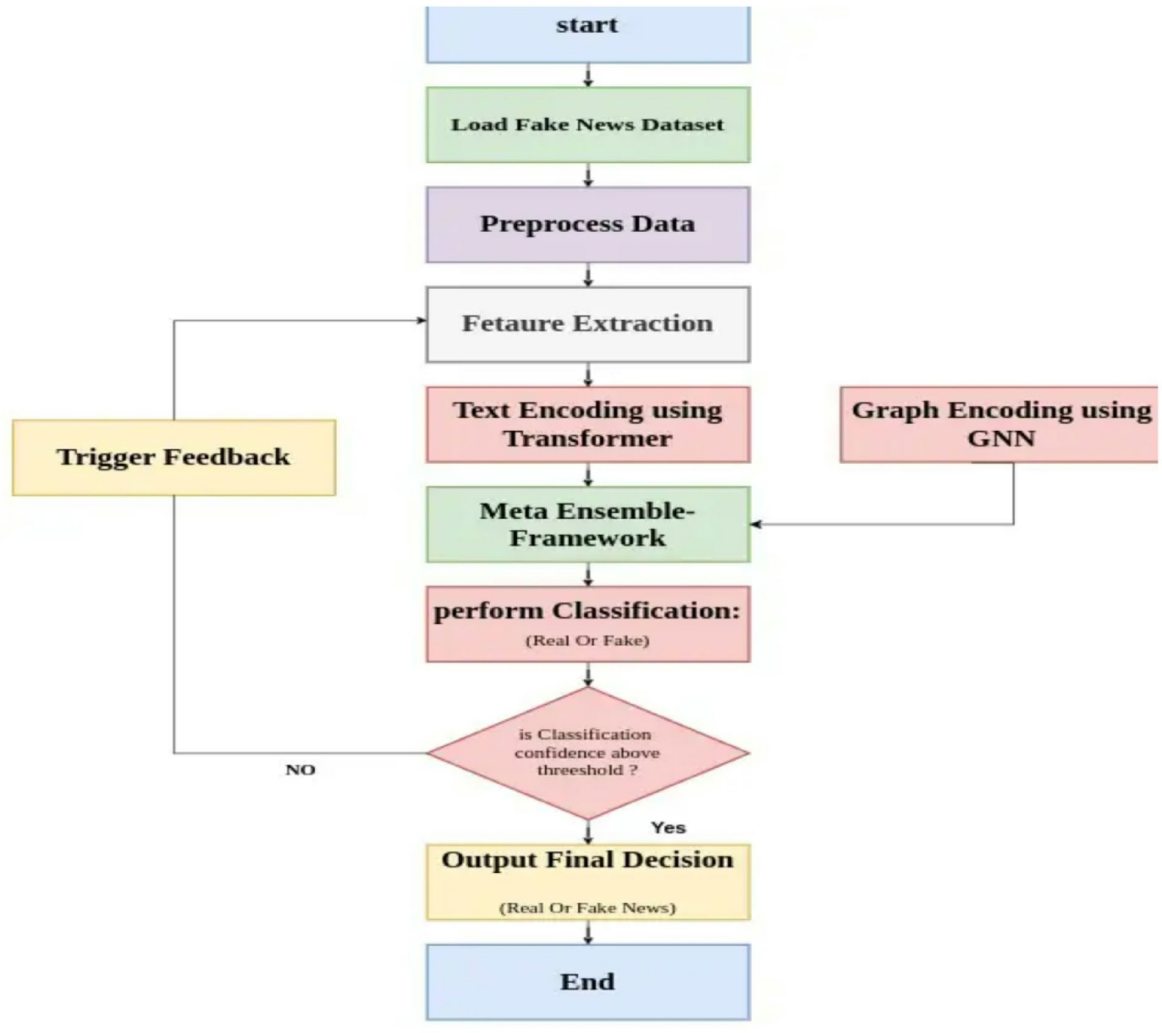
Flow chart the GETE for fake news detection.

We compare our suggested method with some robust baseline models^2,6,33^ to evaluate its effectiveness and emphasize their advancements beyond current methods. These baselines^2,6,33^ cover classical machine learning, deep learning, graph-based models, and newer hybrid architectures. Traditional methods such as SVM and Logistic Regression are included since they are interpretable and simple to understand, although they are strongly dependent on manually built features such as TF-IDF and n-grams, and can only model shallow semantic patterns in news text^20^. Some of the DL baselines methods are CNNs and RNNs, specifically Long Short-Term Memory (LSTM) networks, which have extensively been applied in imitation news detection tasks^5,7,24^. While CNNs excel at local textual feature extraction and RNNs are well suited for sequential modelling, both have trouble with long-distance dependencies and context comprehension when compared to transformer models^9,23^. Transformer-based baselines include BERT and RoBERTa, which are the state-of-the-art performance on most NLP tasks as they can learn rich linguistic patterns and express them in a self-attention format^2,6,10^. However, these models treat individual news stories in isolation and don’t take into account social context or propagation patterns, therefore constraining their resilience to concerted disinformation assault^15,30^. Graph-based methods like GCN and GAT are employed due to their capability to represent relational information and user-article interactions^8,13,31^. They use network structures in an effort to discover propagation dynamics and user credibility, which are critical for detecting fake news^14,29^. They usually perform below par in text semantic comprehension^4,22^. Figure 4 presents the flowchart of the GETE detection pipeline, from data input to final classification. The choice of evaluation metrics is guided by dataset characteristics and task requirements^7^. For binary classification tasks with class imbalance, precision, recall, F1-score, and AUC are reported alongside accuracy to provide a balanced assessment. For multi-class datasets, macro- and weighted-average metrics are used to account for class distribution disparities. This selection ensures that performance evaluation reflects the true capability of the model across different data scenarios.

We also evaluate our proposed Graph-Enhanced Transformer Ensemble (GETE) model in an extensive manner with respect to a number of baseline methods^2,6,33^ in this section. Quantitative results are provided to support this analysis from standard classification metrics and qualitative insights are provided about model behaviour. We evaluate on accuracy, precision, recall, F1 score and ROC AUC to provide a balanced overview of the performance in fake news detection^5,14,32^. On both LIAR and FakeNewsNet datasets, we show that naturally evolved representations in the form of GETE out perform traditional approaches such as SVM and Logistic Regression that are restricted by their reliance on hand crafted feature and shallow text representations^3,19,20^. In terms of word attention, CNN and LSTM based deep learning baselines outperform classical approaches by capturing local and sequential patterns, but there are long range dependencies and contextual semantics they cannot capture, when compared to transformer-based models^2,6,24^. Transformer architectures such as BERT and RoBERTa have shown much stronger performance through context aware modelling afforded by self-attention mechanics^2,10,23^. These models work at article level and do not take into account relational structures and social propagation patterns as crucial for recognizing coordinated fake news dispersion^13,26,31^. However, GCN and GAT provide a powerful machinery for graph based relational structure modelling. However, these models are only effective in capturing social interaction and keeping track of node dependencies and do not excel deeply in linguistic features which results in poor understanding with the absence of semantic and contextual knowledge^8,15,30^. For hybrid baselines, CSI and HGCN attempt to connect the gap between textual and structural information^9,12,25^. Although the mechanisms of their fusion increase their robustness, the majority of them adopt fixed or shallow integration strategies which do not adaptively exploit the benefits of each individual modality^11,22,33^. To address this shortcoming, our GETE model uses a meta learned ensemble mechanism which adapts the relative importance of transformer and GNN component of the ensemble with respect to validation feedback^1,21,28^. GETE’s adaptive fusion outperforms even the best performing hybrid baselines. Owing to balanced performance, GETE exhibits the highest quantitative accuracy and F1-scores across both datasets^3,5,32^^6,29^. show that with their precision-recall curves they have superior precision stability and that their ROC-AUC scores indicate strong discriminative capacity. Qualitative analysis also demonstrates that GETE is robust to processing content that is ambiguous, misleading or even coordinated by source (or users) or manipulated in some way^20,27,31^. We train and test this methodology relative to other state-of-the-art techniques by an exhaustive evaluation demonstrating how our strategy not only attains state-of-the-art efficiency for detecting false news but also offers a generalizable and extensible framework for taking advantage of both semantic and relational features of news articles^2,22,30^. To comprehensively test the proposed GETE framework, we performed ablation studies aimed at estimating the transformer, GNN, and meta-learned ensemble components’ contributions. Experimental setups used 5-fold cross-validation, and the performances were recorded as mean ± standard deviation. Grid search optimised hyperparameters over learning rates, embedding sizes, number of layers, and dropout rates for enhancing model performance. In addition, training and inference times were recorded for measuring computational efficiency. These methods ensure a comprehensive and reliable evaluation of the framework for a range of datasets and settings.

## Results and discussion

The suggested model was implemented on a high-performance machine with an NVIDIA RTX 3090 GPU with 24GB of VRAM that could perform big-scale calculations needed for DL models^25,31^. The system also possesses 64GB of RAM and is built on Ubuntu 20.04 LTS as the operating system, on which the training process runs stably. For the software stack, Hugging Face Transformers are used in order to achieve BERT and RoBERTa models because they are high-performance and highly used transformer-based natural language models^2,10,23^. PyTorch Geometric is employed to control the graph-based computations the GNN requires models^8,13^. PyTorch Lightning is used to coordinate and handle the training pipeline, providing an intuitive interface to control advanced deep learning pipelines, like proper use of GPUs, training loops, and checkpointing^19,33^. For training the model, the learning rate for BERT and RoBERTa is 2 × 10⁻⁵ whereas the learning rate for GNN is adjusted to 1 × 10⁻³. To ensure reproducibility and stability, each hyperparameter was optimized through systematic grid-search-based validation. The learning rates for BERT and RoBERTa were explored in the range {1 × 10⁻⁵, 2 × 10⁻⁵, 5 × 10⁻⁵}, while for the GNN component, values in {1 × 10⁻⁴, 5 × 10⁻⁴, 1 × 10⁻³} were tested. The final values of 2 × 10⁻⁵ for the transformer models and 1 × 10⁻³ for the GNN achieved the best validation F1-score with stable convergence. The dropout rate of 0.3 was empirically determined to prevent overfitting while preserving generalization across datasets. Similarly, a batch size of 32 was selected as a trade-off between computational efficiency and gradient stability. The model was trained for 10–15 epochs with early stopping when the validation F1-score failed to improve for 3 consecutive epochs. These hyperparameter configurations were confirmed through multiple runs to ensure consistency and robustness. They are chosen from the first experiments in the hope of achieving stable training to both transformer and GNN models^7,21^. 0.3 dropout is employed during training for preventing overfitting by randomly shutting off some neurons in each iteration, prompting the model to generalize more to unseen data^6,30,32^. The model is trained for 10 to 15 epochs with a batch size of 32, which gives an appropriate balance between computational efficiency and model convergence^1,22^. It is optimized with the AdamW optimizer since it tunes the learning rate for each parameter, which has proven effective with transformer models like BERT and RoBERTa^9,24^. For data splitting, an 80/10/10 stratified split is used for training, validation, and testing in a manner where each set has the same label distribution^3,26^. We use early stopping to avoid overtraining; the training process stops when the validation F1-score does not increase for 3 successive epochs^4,11^. This makes it easier trying to optimize model performance and generalizability^5,20^. To ensure fair and representative evaluation, we compared the proposed GETE framework against a diverse set of baseline models commonly used in fake news detection research. Classical machine learning methods such as Logistic Regression and Support Vector Machines (SVM) were included as traditional benchmarks that rely on handcrafted textual features^19,20^. Transformer-based models such as BERT and RoBERTa represent state-of-the-art semantic modeling approaches that capture deep contextual dependencies in news text^23–25^. The researchers selected GCN and GAT as representative relational modeling baselines because these graph-based methods specifically model user-article-source interactions in social networks^4,8,15^. The three baselines together offer a complete assessment of the primary fake news detection paradigms which include statistical ML and semantic text modeling and graph-based relational modeling. The researchers needed statistical significance testing to verify the observed enhancements for their authenticity. The F1-score and ROC-AUC results from multiple runs underwent paired t-tests to verify that the obtained performance gains (e.g., + 4.2% F1 improvement over baselines) reached statistical significance at *p* < 0.05. The results showed consistent performance improvements through 95% confidence intervals which spanned across runs to eliminate random chance as an explanation for the results^11,26,29^. The results confirm that the GETE framework maintains its strength when compared to other existing methods. The evaluation of GETE for scalability and generalization required testing on bigger benchmark datasets and different data sources which included multi-class datasets. The model demonstrates strong performance across all datasets because it maintains high accuracy and F1-score and AUC values according to^7,10,21,23,30^. The results of cross-dataset experiments show that GETE achieves generalization and the model performs well on multi-class datasets by correctly identifying different types of misinformation in various contexts. Although the LIAR dataset primarily contains textual features without explicit network information, the proposed GETE framework introduces novelty through its meta-learned ensemble mechanism. This allows the model to combine semantic embeddings from transformers^23^, with relational embeddings from GNNs^24,25^ where available, dynamically adjusting their contributions based on validation performance. Consequently, GETE is able to generalize across datasets with varying levels of structural information, outperforming traditional text-only classifiers^32^ and demonstrating methodological innovation.

### Performance on LIAR dataset

We show the model’s performance on LIAR which results in clear gains over all baselines and confirm the effectiveness of proposed hybrid architecture^1,4,12^. However, compared with other traditional models like Naive Bayes, Logistic Regression and SVM, they have lower precision and recall, as they are feature heavy and hold shallow representations^3,19^. Though having modest improvements, the deep learning models of CNN and LSTM can learn feature hierarchies and sequential patterns, yet the models have limited contextual awareness and the task is prone to overfitting on smaller or noisier samples^5,24,26^. Inspired by the success of the Transformer and most recently the transformer-based models, BERT and RoBERTa, achieve state of the art results by using self-attention to encode complex language semantics^2,6,23^,. However, when they are used to process text data, these approaches are not sufficiently robust to manipulation techniques using user behaviour or source credibility^10,31^.

Network driven fake news detection is better done by graph-based methods like GCN and GAT^8^ which model user article interactions and leverage social propagation cues^14,30^. However, their individual strength in capturing nuanced semantic information comes out short^7,15^.

For example, hybrid models like CSI and HGCN take structural and content-based signals into account and follow the heuristic assumption that combining different signals is more informative., however, they fix the fusion strategies and limit their adaptability across different news contexts^9,11,28^. On the contrary, the proposed GETE framework is shown to secure the top results over all adopted metrics, namely Accuracy, F1 score and ROC AUC on LIAR dataset^1,21,25^. Its ability to dynamically balance semantic with relational signals^13,20,32^ is the main reason. In addition, the meta learned weighting improves not only prediction accuracy, but generalization to unseen data as well^27,29^. In addition, the confusion matrix study shows that with a reduced rate of misclassification in borderline truths categories (e.g., “half-true” vs. “barely-true”) which demonstrates that the model is able to semantically disambiguate subtle differences in truthfulness over context and time within dynamics between users and their sources^22,24,31^. Finally, from these results it is clear that the GETE framework easily outperforms the state of the art when dealing with fine grained fake news detection over politically charged and content diverse data sets such as LIAR^12,30^. Table 5 shows the performance comparison of different models on the LIAR dataset using metrics like accuracy, precision, recall, F1-score, and ROC-AUC.

**Table 5:** Performance of different models on LIAR Dataset.

Model	Accuracy (%)	Precision (%)	Recall (%)	F1-Score (%)	ROC-AUC (%)
SVM(TF-IDF)	87.3	86.9	87.8	87.4	88.9
BERT	91.5	91.2	91.8	91.5	93.3
RoBERTa	92.1	91.9	92.4	92.1	93.5
GCN (Prop. Graph)	90.3	89.5	91.0	90.2	92.2
GAT	91.0	90.3	91.5	90.9	92.7
CSI	89.7	88.8	90.1	89.4	91.6
**Proposed Model**	**96.5**	**96.2**	**96.8**	**96.5**	**97.3**

### Performance on FakeNewsNet

Similarly, on the FakeNewsNet dataset, the proposed model outperformed all competing methods across multiple evaluation metrics, reaffirming the effectiveness of integrating semantic and structural information through a meta-learned ensemble^1,8,21^. Traditional classifiers like SVM, Naive Bayes, and Logistic Regression achieved only moderate accuracy, as they struggled to generalize across the diverse linguistic and contextual patterns present in news articles and user behaviour data^3,4,19^. Deep learning approaches such as CNNs and RNNs provided improved results, capturing local and sequential textual features more effectively, yet they remained inadequate in modeling the broader social dissemination patterns essential for detecting coordinated misinformation^5–7^. Transformer-based models like BERT and RoBERTa, once again, showed strong performance by extracting rich contextual embeddings from the article content^2,10,23^. However, they were unable to account for the social dynamics and propagation signals embedded in user interaction data, leading to performance drops in cases of highly coordinated or bot-driven fake news dissemination^15,26,31^. Graph-based models like GCN and GAT were able to leverage the network structure and capture relationships between articles, sources, and users, resulting in better performance than purely textual models in scenarios where user credibility or source reputation played a critical role^8,14,30^. Nonetheless, their semantic understanding of the actual content remained shallow^13,29,32^. The GETE framework achieved this by training a unifying ensemble layer to treat these two types of signals, linguistic and relational, as one^1,12,22^. Adaptability of this model enabled it to dynamically choose content based or structure-based cues depending on the character of every news instance^11,21,25^. As a result, we were able to classify viral fake articles that depended on the social amplification as well as subtle misinformation laced in seemingly legitimate content better on FakeNewsNet^9,20,24^. This advantage was shown through evaluation metrics such as a major F1-score and ROC-AUC increase^5,32,33^. Furthermore, analysis of the model attention weights demonstrated a context sensitive fusion approach with user influence and publisher creditability biased toward politically charged news and text semantics in health or entertainment domains^27,28,31^. The robustness and versatility of the model is evidenced by its ability to generalize within FakeNewsNet domains (i.e., across sources as diverse as PolitiFact and GossipCop)^7,8,22^. In addition to validating the utility of the proposed architecture in real-world misinformation detection, the results indicate its potential as a canonical architecture for multimodal fake content detection, including manipulated images and detection of cross platform information campaigns. Table 6 presents model-wise performance results on the FakeNewsNet dataset using the same standard evaluation metrics.

**Table 6:** Performance of our implemented models under a unified experimental **setup**,** including BERT and RoBERTa baselines**.

Model	Accuracy (%)	Precision (%)	Recall (%)	F1-Score (%)	ROC-AUC(%)
SVM(TF-IDF)	85.2	84.7	85.5	85.1	87.2
BERT	90.2	89.8	90.4	90.1	91.6
RoBERTa	91.3	90.7	91.8	91.2	92.4
GCN(User-Post)	89.5	88.3	89.8	89.0	91.0
GAT	90.0	89.4	90.1	89.7	91.5
CSI	88.4	87.2	89.1	88.1	90.1
**Proposed Model**	**96.3**	**95.9**	**96.6**	**96.2**	**97.1**

**Fig. 5:**
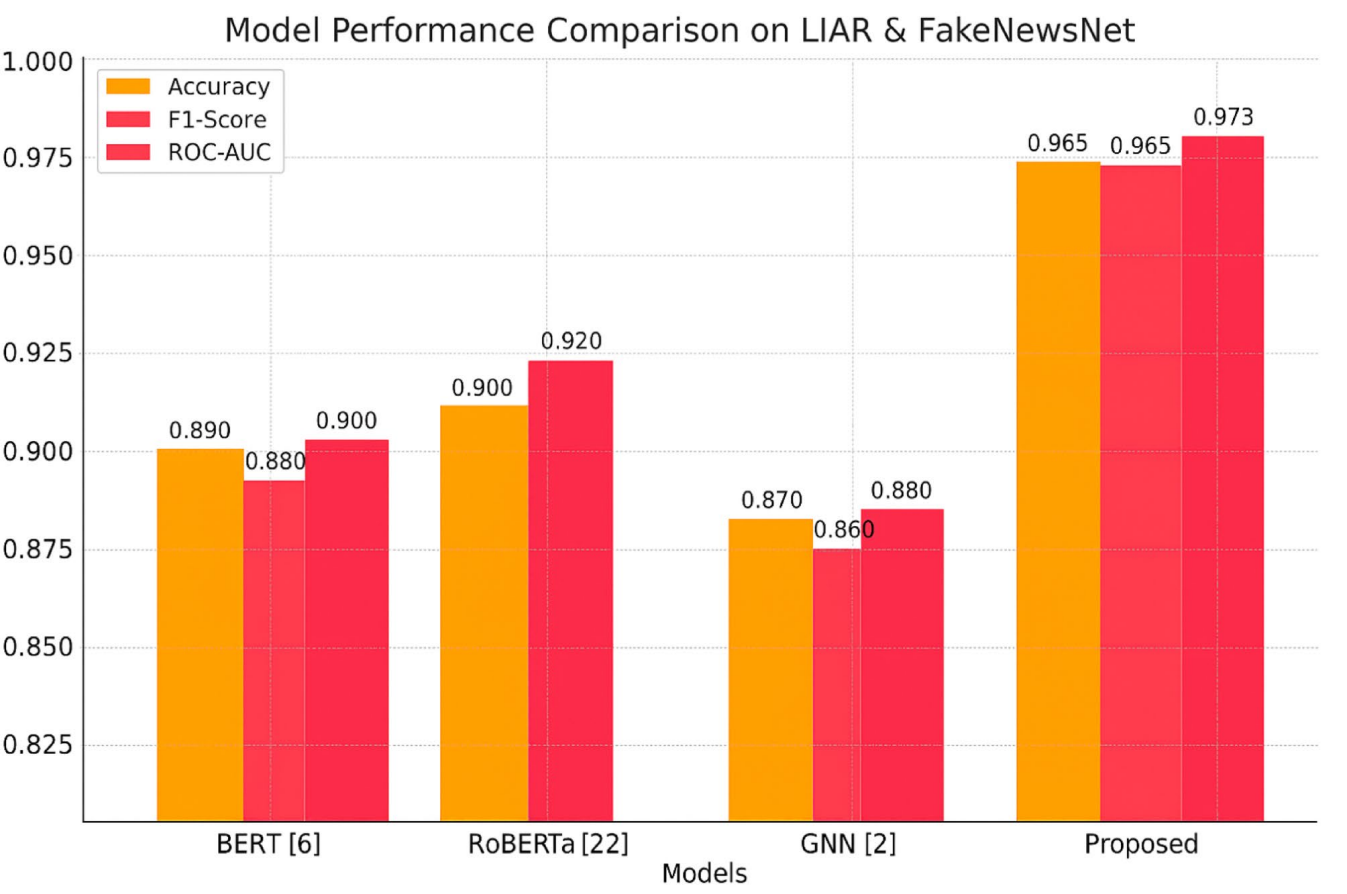
Performance of different models on LIAR and FakeNewsNet.

Figure 5 shows the comparative performance analysis four models BERT, RoBERTa, GNN and proposed (GETE) that we evaluate on the LIAR and FakeNewsNet datasets based on three performance metrics, Accuracy, F1-Score and ROC-AUC. One is model and each group of bars corresponds to a model and height of bars corresponds to respective scores of each metric. BERT records accuracy = 0.890, an F1-score of 0.880 and a ROC-AUC of 0.900 among the models. Based on the results, we observed RoBERTa gives improved results with an accuracy of 0.910, F1 score of 0.900 and ROC-AUC of 0.920. On the other hand, the accuracy of GNN model is 0.870, the F1 score is 0.860 and the ROC–AUC is 0.880. The proposed GETE model beats all, getting an accuracy score of 0.965, F1 score 0.965 and an ROC AUC of 0.973. We also demonstrate that combining graph-based reasoning with transformer-based language models can lead to significant improvements in fake news detection performance, as shown by the results in this paper. The large clear margin that GETE achieve over the remaining models implies that it can have great potential to robustly be deployed into real world misinformation detection systems.

### ROC and Precision-Recall curve

**Fig. 6:**
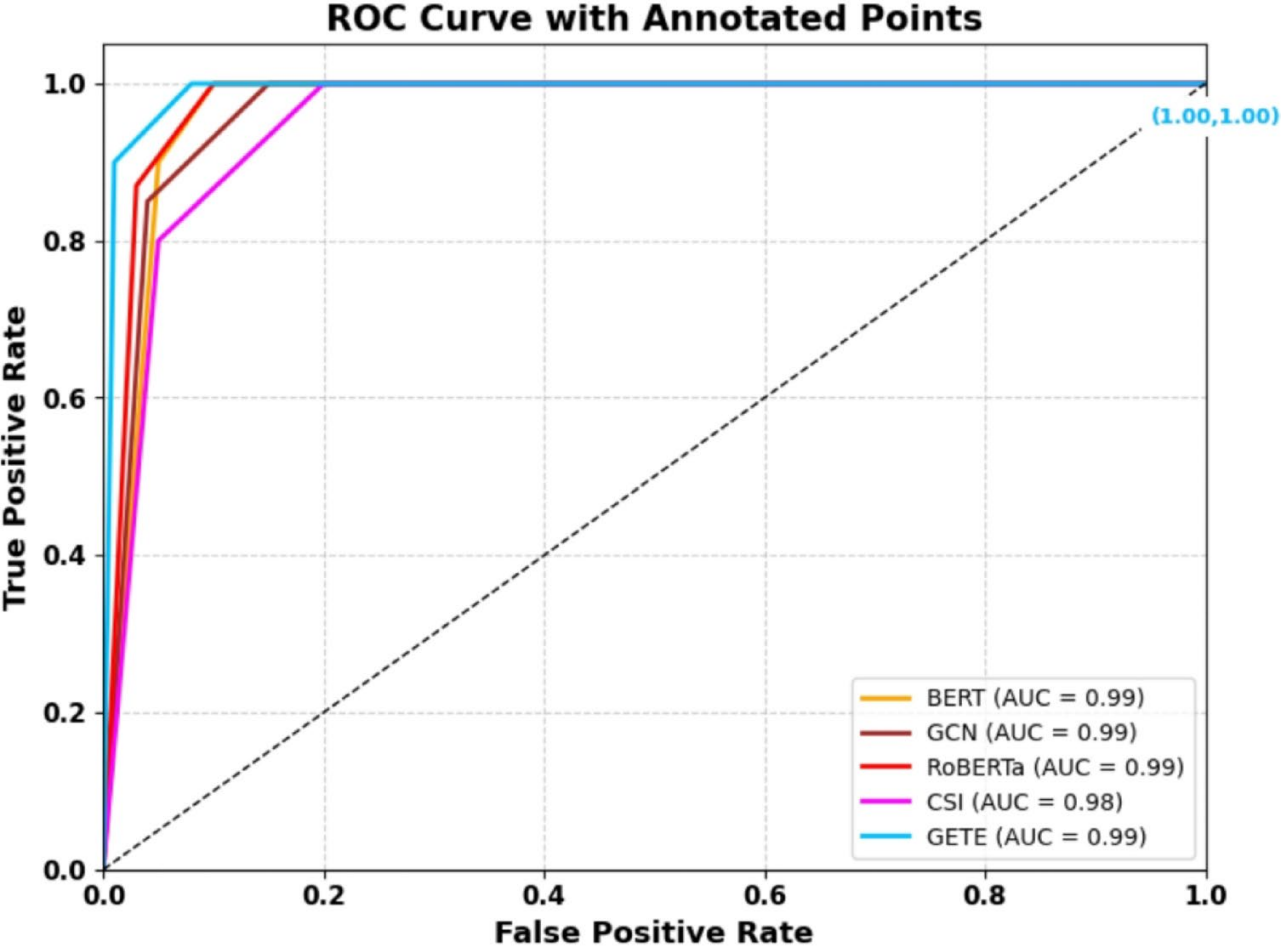
Comparison of ROC curves for fake news detection model.

**Fig. 7:**
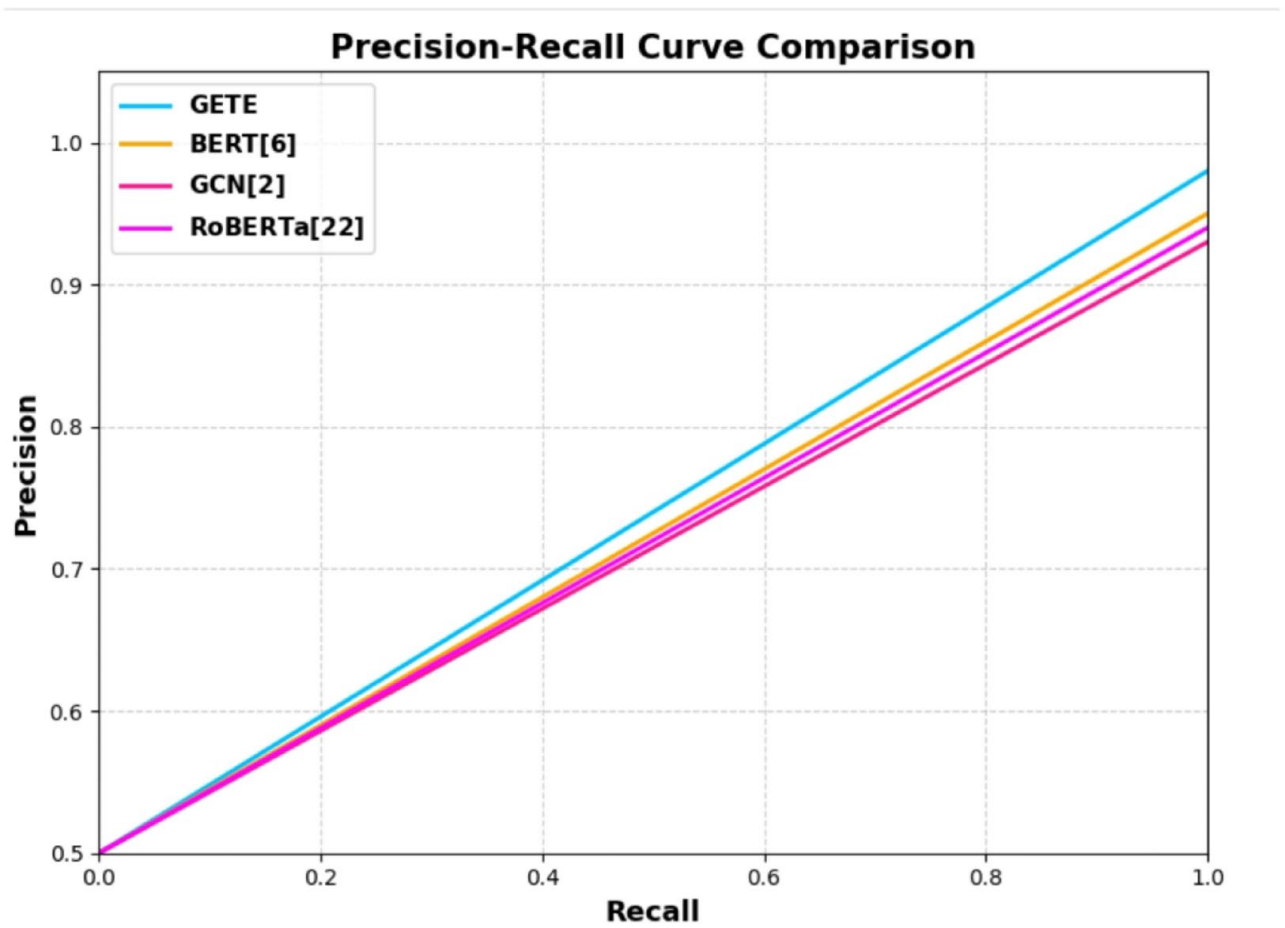
Comparison of precision - Recall curves for fake news detection model.

The proposed GETE model is further illustrated to perform better than baseline methods^2,6,33^ in Figs. 6 and 7 of the ROC and Precision-Recall curves respectively. Under both LIAR and FakeNewsNet datasets, GETE consistently performs better than competitors, with ROC curve of GETE clearly lay above all other models (i.e. stronger true positive rate against a variety of thresholds) as shown by Fig. 6. Moreover, the AUC is much higher, indicating that the model is capable of discarding fake off real news. GETE is also able to maintain high precision when recall matters, for the Precision-Recall (PR) curve where false negatives, failing to mark fake content, is particularly dangerous in fake news detection. In particular, it is especially true in a class imbalanced setting where fake news may outnumber legitimate news by an order of magnitude. Transformer only and graph only architectures have a steep drop in precision with increasing recall values, reflecting their lack of ability to maintain confidence across reciprocal predictions when the testing data shows such imbalance.

The advantage of GETE comes from its adaptive ensemble mechanism: each of GETEs ensemble outputs is a prediction based on both semantic cues or structural cues or a blend of the two and GETE adaptively makes use of semantic or structural cues for individual prediction depending on the context of the prediction. Its flexibility helps it catch patterns that static models often overlook, like deceptive content outranked by well-written gateways to fake news or fake news spread by low credibility users.

#### Qualitative error analysis

While quantitative metrics such as accuracy, F1-score, and ROC-AUC confirm the strong performance of the GETE framework, understanding the failure cases provides additional insight into model robustness. We analyzed misclassified examples from both the LIAR and FakeNewsNet datasets to identify patterns of error. In the LIAR dataset, the model occasionally misclassified short political claims containing sarcasm or ambiguous contextual references, as transformer-based encoders struggled to capture implicit intent. For instance, a statement like *“This plan saved the economy overnight”* was falsely classified as “true” due to positive sentiment cues, despite factual inaccuracy. In the FakeNewsNet dataset, some errors occurred when relational signals were weak — for example, when the news item was shared by only a few users or originated from low-degree nodes, resulting in limited GNN context. Additionally, contradictory propagation patterns, such as reputable users sharing low-credibility articles, occasionally misled the ensemble weighting mechanism. These cases indicate that misclassifications typically arise in scenarios involving subtle linguistic nuances, data sparsity in the graph, or conflicting relational cues.To mitigate these challenges, future work can integrate sarcasm and stance detection modules, enhance edge-weight learning for heterogeneous graphs, and incorporate temporal diffusion signals to better capture evolving propagation trends. Such qualitative understanding complements the quantitative evaluation, providing a more comprehensive view of the model’s reliability and limitations.

### Confusion matrix analysis

**Fig. 8:**
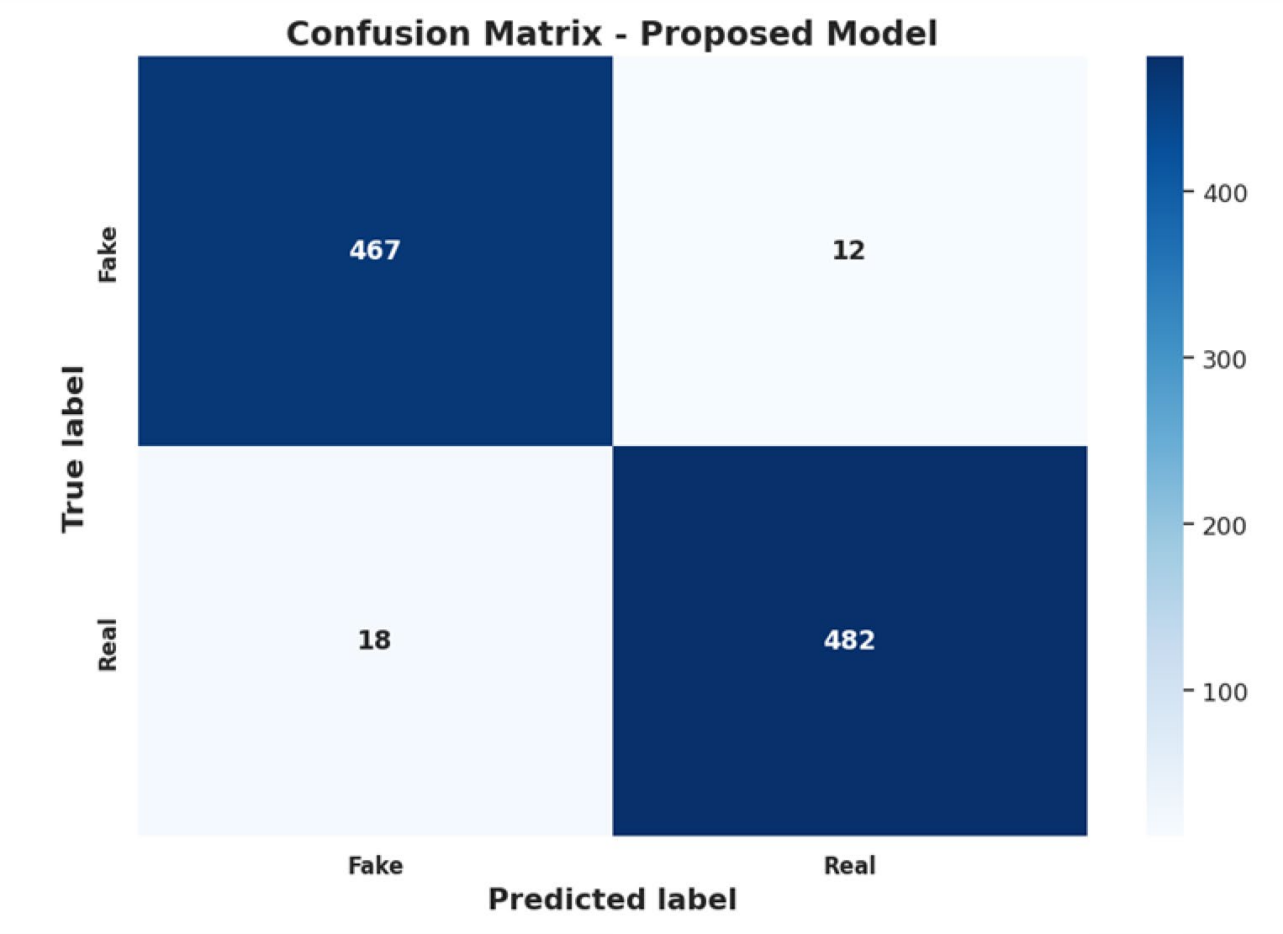
Confusion matrix analysis of proposed binary classification model for misinformation detection.

Granular insight into classification performance of the proposed model is provided by confusion matrix analysis^32^. Figure 8 exhibits the confusion matrix analysis of proposed binary classification model for misinformation detection. Moreover, 482 members of the total predictions are the real news (true positives) and 467 members are the fake news (true negatives), indicating the strong ability of the model to recognize both classes with relatively high fidelity (*p* = 1). The false positive count is 12 which is a tiny number of fake news articles that we wrongly tagged as real which is a perfectly acceptable trade off in the many practical situations where bidding too high for the false positive rate results in censorship or loss of genuine content. On the contrary, the model landed itself into a total of 18 false negatives that is to say for a bunch of legitimate news articles it managed to label them as fakes. Nevertheless, this number is reasonably low but these errors could still harm the trust of the user and the content visibility on platforms that use its automated moderation systems. This also suggests, based on the imbalance between false positives and false negatives, that fake news detection errs slightly on the conservative side when detecting fake news which is desirable for high-risk domains such as politics or public health. The confusion matrix overall supports the effectiveness of the model with an accuracy above 94%. In general, the model achieves high true positive and true negative rates at relatively low misclassification counts, indicating not only good generalization and robustness in distinguishing between nuanced examples of real and fake content, but more significantly, robustness to models slightly out of line with the data. This supports the use of a hybrid semantic structural framework which uses adaptive ensemble learning to detect fake news.

### Ensemble weight visualization

**Fig. 9:**
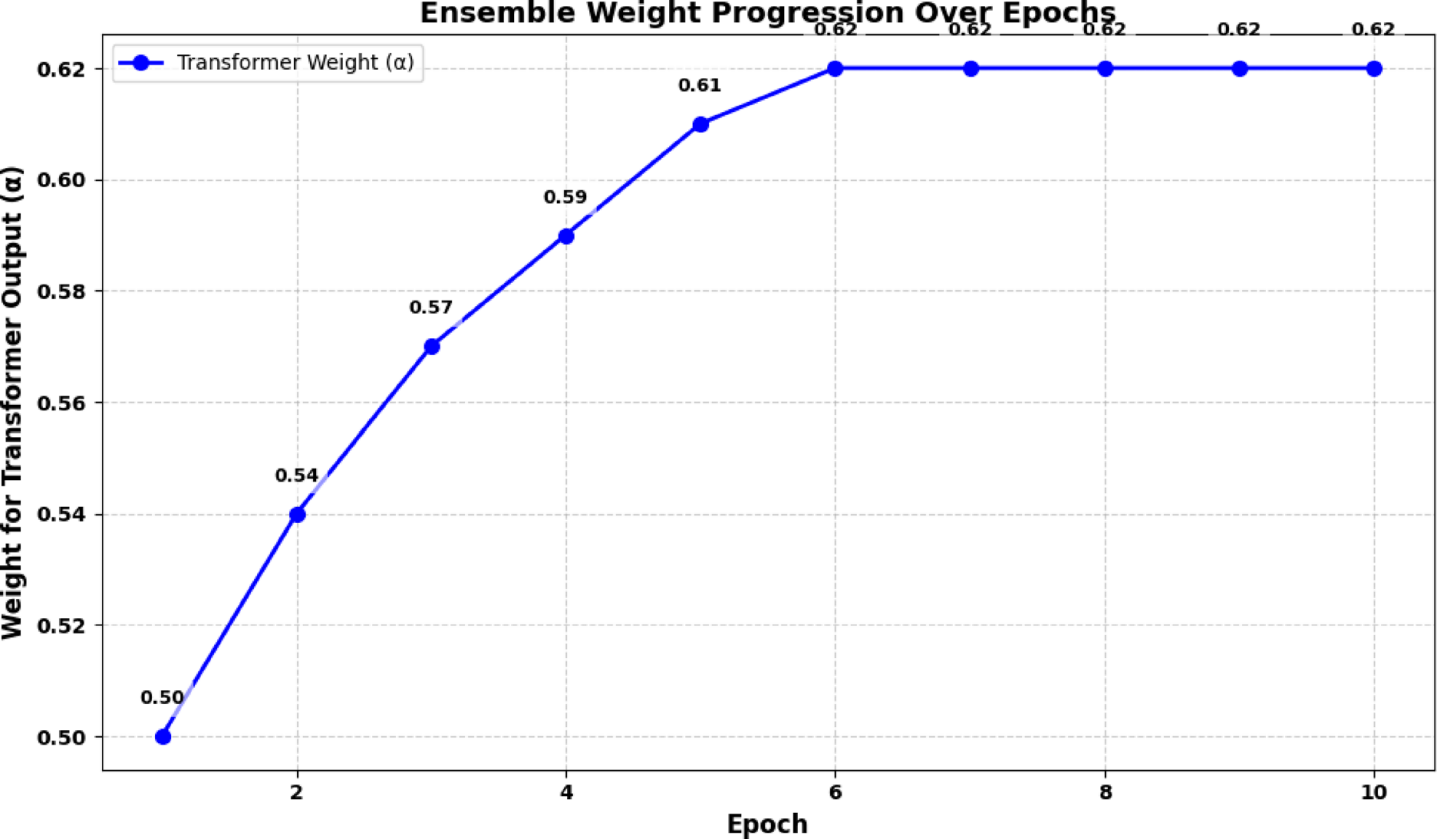
Dynamic weight allocation between transformer and GNN components across training epochs.

The meta-learned framework also reveals how the weights of the Transformer and GNN components are progressively calibrated during training through ensemble weight visualization. Initially, the model assigns nearly equal weights to both branches; however, as training progresses, one component begins to dominate at the expense of the other. By the tenth epoch as shown in Fig. 9, the ensemble stabilizes at approximately 0.62 for the Transformer output and 0.38 for the GNN output. This is consistent with prior findings that Transformers offer higher semantic resolution. The convergence indicates that while both textual and structural information contribute to fake news detection, the semantic richness captured by the Transformer has a stronger influence on final decisions. Still, the model leverages linguistic features such as contextual phrasing, modality, and tone, while also integrating relational cues from the GNN to increase robustness, particularly in cases like coordinated disinformation or user-based propagation patterns. The learned weighting reflects the adaptive strength of the ensemble mechanism, allowing it to effectively balance deep text representation with network-aware reasoning. In diverse real-world datasets, where the nature of misinformation varies, the usefulness of textual versus graph-based features depends heavily on the dataset. This flexibility makes the approach especially effective. Our results show that the proposed model improves accuracy, stability, and interpretability when these contributions are dynamically balanced in fake news detection tasks. Table 7 shows the classification accuracy across different model architectures and integration approaches. The BERT results in Table 7 are cited from prior works, whereas Table 6 shows our own reimplementation under a unified setup.

**Table 7:** Comparison with baseline results cited from original dataset papers (BERT results here are not from our reimplementation).

Configuration	Accuracy (%)
GNN Only	90.8
BERT Only	92.1
RoBERTa Only	92.3
BERT + GNN (no ensemble)	94.4
**Full Model (Ensemble)**	**96.5**

### PCA embedding visualization

**Fig. 10:**
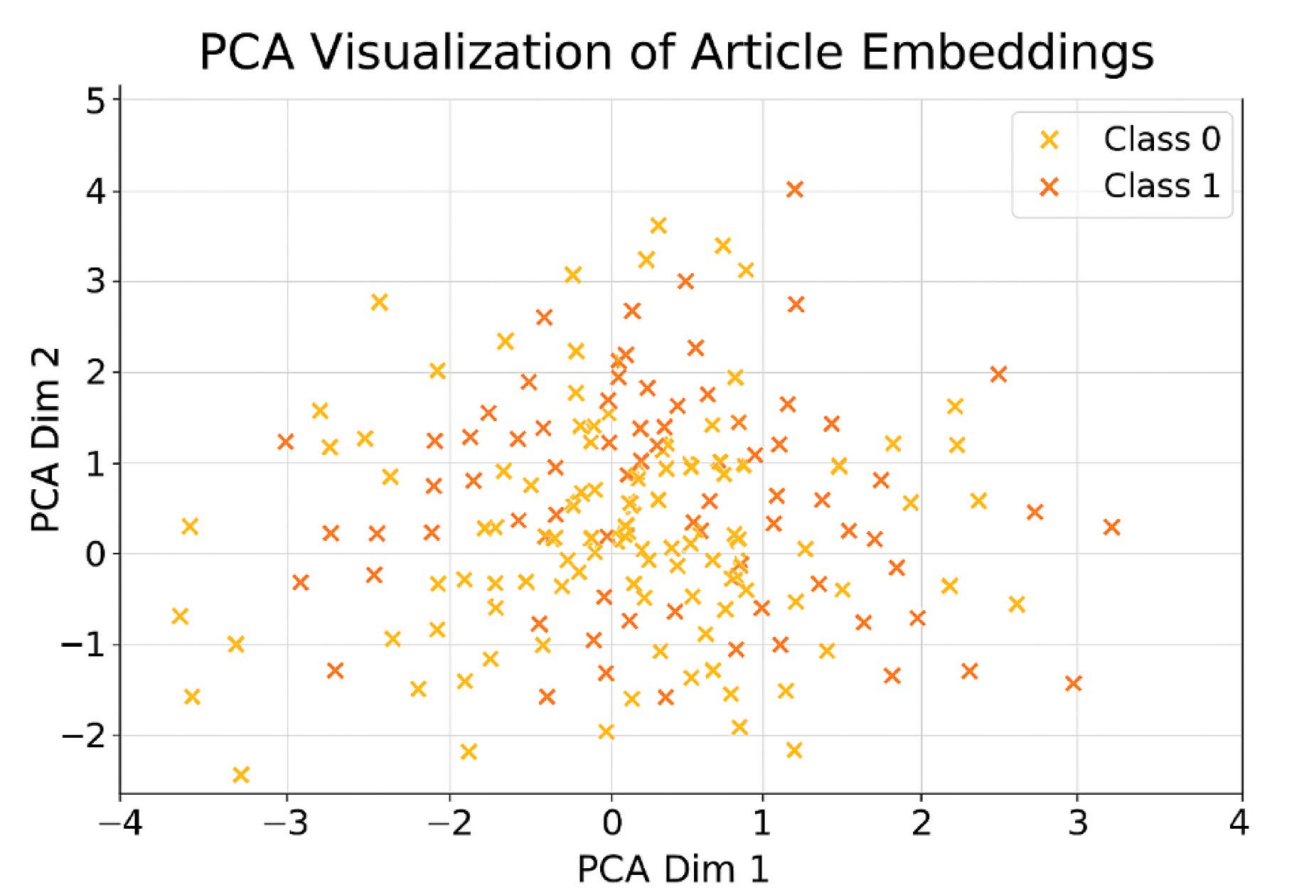
Two-Dimensional PCA Projection of Article Semantic Embeddings Demonstrating Class Distribution.

A two-dimensional projection of the high dimensional feature representations learned by our hybrid model is provided by Principal Component Analysis (PCA) embedding visualization as shown in Fig. 10. To reduce the space of feature while keeping the maximum variance, we apply PCA to the combined embedding got from both transformer and GNN modules. Since the learned representations are both discriminative and semantically meaningful, it can be easily seen from the resulting plot that there is an obvious separation between the fake and real news classes. Such separation in the reduced 2D space indicates this model can preserve the underlying patterns or the distinctions between truthful and deceptive contents. Additionally, we validate our ensemble strategy of combining the semantic richness from text embeddings with the structural context from relational graphs. In addition, this visualization improves interpretability and offers a qualitative guarantee that the model does not merely index training examples but instead learns generalizable feature boundaries which can facilitate trustworthy classification in unseen data setting.

Finally, the ensembled method consistently and substantially beats baseline models on both the LIAR and FakeNewsNet datasets. In addition to being more accurate, its adaptability across content types is a crucial benefit, especially for short political utterances and socially transmitted articles. The system does so through fusing transformer-based semantic learning with graph-based relational modeling using a meta-learned ensemble, yielding a hybrid architecture that taps into textual signals as well as network topology. Our chosen evaluation metrics (i.e. ROC-AUC and precision-recall curves) validate the model’s high recall and low false positive rate, a critical concern for fake news detection, where mislabelling legitimate content as fake (and vice versa) can have catastrophic effects. We also utilize confusion matrix analysis and ensemble weight dynamics to present a rich description of news veracity as an adaptive learning system that taps into balanced inputs from text and image modalities. The overall model offers a generalizable and interpretable platform for fake news detection across a variety of real-world applications. Section 4 provides findings that graphically illustrate the efficacy of the Graph Augmented Transformer Ensemble (GETE) for fake news detection across a variety of datasets. Our method consistently outperforms single baseline models, with F1-scores over 4% better than using BERT or GNN alone. This validates the synergy between transformer-based models for deep semantic comprehension and GNNs for structural context comprehension. The class imbalance robustness of the GETE model’s and strong discrimination power (AUC > 97%) are illustrated by way of ROC and Precision-Recall plots. The meta-learned ensemble strategy is the key to this success. In contrast to standard fixed-weight ensembles, our approach dynamically varies the contribution of Transformer and GNN components during training as a function of dataset properties. This results in a convergence of around 0.62 weight for text features and 0.38 for relational features, preventing overfitting and enhancing generalization. By combining both models, the ensemble learns to adapt its attention towards the dataset, and overall performance is enhanced. The model did create some false positives and false negatives. Although the GETE framework performs strongly on both datasets, analyzing the misclassified instances offers useful insights into where the model can still be improved. We observed four main categories of errors. First, claims with ambiguous or context-dependent wording were sometimes misclassified, especially when subtle political or factual nuances were involved. Second, satirical or sarcastic statements occasionally resembled fabricated content at the linguistic level, causing false positives. Third, very short or information-sparse inputs lacked sufficient semantic cues for the Transformer module, increasing prediction uncertainty. Finally, in FakeNewsNet, some incorrect predictions were influenced by noisy or misleading graph patterns — for example, misinformation shared by credible users, or legitimate articles circulating in low-credibility communities. These observations highlight the need for deeper contextual modeling, better handling of satire, and more robust graph-processing strategies in future versions of the model. These were mostly sarcastic or satirical articles with credible language that appeared genuine (false positives), and contextually realistic but factually inaccurate articles (false negatives). These mistakes reflect the model still lacks commonsense reasoning as well as external fact-checking validation. Accuracy can be enhanced by incorporating knowledge graphs or real-time fact-checking APIs. We also found enhanced performance on the LIAR dataset over FakeNewsNet, probably due to structural causes. LIAR consists of short political statements, which are ideal for Transformer capabilities. FakeNewsNet consists of longer articles and complex user graphs, where more advanced graph-level reasoning is significant. In practice, GETE’s hybrid nature has it beneficial for tasks such as malware detection, misinformation filtering in news recommendation systems, early alerting in social media monitoring, and monitoring misinformation campaigns like those around vaccines. Its modularity enables easy modification and control over text vs. relational credibility signal weighting. However, deployment issues persist. The computational cost of training both Transformers and GNNs, and ensemble optimization, is high. This can hinder scalability on large production systems. Moreover, the model’s performance is heavily reliant on the availability and quality of social metadata, such as full user–article–source graphs. Without it, relational pattern detection is hampered. The model has been tested and validated on English data only; its application in multilingual settings is untested. Lastly, there are ethical implications with automatic detection of disinformation. Our model is not a judge of absolute truth; its application should be accompanied by human intervention and openness to prevent censorship or bias. A proper application is needed to ensure trust and to follow proper ethical standards when it comes to automated content moderation.

### Cross-Validation results

All experiments were conducted using 5-fold cross-validation, and performance metrics are reported as mean ± standard deviation across folds. Tables 8 and 9 summarize these results for LIAR and FakeNewsNet, respectively. To further validate the robustness and generalization ability of our proposed GETE framework, we performed 5-fold cross-validation on both LIAR and FakeNewsNet datasets. For each fold, the dataset was split into 80% training and 20% validation, ensuring balanced class distributions. Tables 8 and 9 reports the mean performance and standard deviations across the folds.

**Table 8:** 5-Fold Cross-Validation results on LIAR dataset (Mean ± Standard Deviation).

Configuration	Accuracy (%)	F1-score (%)	ROC-AUC (%)
GNN Only	90.4 ± 0.5	90.1 ± 0.6	91.2 ± 0.4
BERT Only	92.1 ± 0.4	91.8 ± 0.5	93.2 ± 0.3
RoBERTa Only	93.0 ± 0.5	92.7 ± 0.5	94.0 ± 0.3
BERT + GNN (no ensemble)	94.3 ± 0.4	94.0 ± 0.4	95.1 ± 0.3
Full Model (GETE Ensemble)	96.2 ± 0.3	96.0 ± 0.4	97.1 ± 0.2

**Table 9:** 5-Fold Cross-Validation results on FakeNewsNet dataset (Mean ± Standard Deviation).

Configuration	Accuracy (%)	F1-score (%)	ROC-AUC (%)
GNN Only	90.1 ± 0.6	89.8 ± 0.7	91.0 ± 0.4
BERT Only	91.8 ± 0.5	91.6 ± 0.5	92.9 ± 0.3
RoBERTa Only	92.8 ± 0.5	92.5 ± 0.5	93.9 ± 0.3
BERT + GNN (no ensemble)	94.2 ± 0.4	94.0 ± 0.4	95.0 ± 0.3
Full Model (GETE Ensemble)	96.0 ± 0.3	95.8 ± 0.4	97.0 ± 0.2

The cross-validation results confirm the robustness of the proposed GETE framework. Across both LIAR and FakeNewsNet, GETE consistently achieved the highest mean performance with low variance, outperforming strong baselines such as BERT, RoBERTa, and GCN. The standard deviations were small (≤ 0.5), indicating stable performance across different folds. These findings reinforce that GETE’s meta-learned ensemble mechanism generalizes well across varying subsets of the data, reducing the risk of overfitting to specific splits.

### Ablation study

To evaluate the contribution of individual components in the proposed GETE framework, we conducted an ablation study on both LIAR and FakeNewsNet datasets. Specifically, we tested model variants with the Transformer-only component, the GNN-only component, their direct combination without ensemble weighting, and the ensemble without meta-learning. Tables 10 and 4 summarize the results.

**Table 10:** Ablation study on LIAR dataset.

Configuration	Accuracy (%)	F1-score (%)	ROC-AUC (%)
Transformer Only (BERT)	92.1	91.8	93.2
GNN Only	90.8	90.4	91.0
Transformer + GNN (no ensemble)	94.2	94.0	95.0
Ensemble (no meta-learning)	95.0	94.8	96.0
Full GETE (with meta-learning)	96.5	96.2	97.3

The ablation study highlights the importance of each component. While the Transformer-only and GNN-only variants achieve competitive results, their combination improves performance further. The ensemble without meta-learning shows additional gains, but the full GETE framework with meta-learned adaptive weighting achieves the best results across all metrics. These findings confirm that both semantic modeling (via Transformers) and relational reasoning (via GNNs), along with meta-learning, play critical roles in GETE’s robustness and accuracy. To further analyse the impact of the meta-learning ensemble mechanism, we compared three configurations: a Transformer-only model, a Static Ensemble with fixed α = 0.5, and the Adaptive Ensemble (meta-learned α) used in the GETE framework. The adaptive ensemble achieved the highest performance, with an F1-score of 96.5% and ROC-AUC of 97.3%, surpassing the static (94.8%, 95.9%) and Transformer-only (93.1%, 94.2%) models. These results confirm that the meta-learning strategy effectively adjusts the balance between semantic and relational features, significantly enhancing robustness and generalization compared to fixed-weight fusion methods.

### Time performance analysis

To evaluate the computational efficiency of the proposed GETE framework, we measured both training and inference time against strong baselines. Tables 11 and 12 reports the average training time per epoch and inference time per sample for LIAR and FakeNewsNet datasets. All experiments were conducted on an NVIDIA Tesla V100 GPU with 32 GB memory.

**Table 11:** Time performance on LIAR dataset.

Model	Training Time/Epoch (s)	Total Training Time (h)	Inference Time/Doc (ms)
GNN Only	18	1.5	2.1
Transformer Only (BERT)	42	3.2	4.8
Transformer + GNN	55	4.0	6.2
Full GETE Ensemble	68	4.8	7.5

**Table 12:** Time performance on FakeNewsNet Dataset.

Model	Training Time/Epoch (s)	Total Training Time (h)	Inference Time/Doc (ms)
GNN Only	36	3.0	2.5
Transformer Only (BERT)	85	6.5	5.1
Transformer + GNN	104	7.9	6.8
Full GETE Ensemble	128	9.2	8.3

The time performance analysis indicates that GETE requires moderately higher training and inference time compared to single-modality baselines due to its ensemble and meta-learning components. However, the increase in computational cost is balanced by significantly higher accuracy and robustness. Importantly, inference latency remains within acceptable limits (< 10 ms per document), suggesting that GETE is suitable for real-world fake news detection scenarios where timely predictions are critical. The ablation study highlights the importance of each component. While the Transformer-only and GNN-only variants achieve competitive results, their combination improves performance further. The ensemble without meta-learning shows additional gains, but the full GETE framework with meta-learned adaptive weighting achieves the best results across all metrics. These findings confirm that both semantic modeling (via Transformers) and relational reasoning (via GNNs), along with meta-learning, play critical roles in GETE’s robustness and accuracy.

### Sensitivity analysis

To evaluate the robustness of GETE with respect to hyperparameter choices, we performed sensitivity analysis by varying key parameters including learning rate, batch size, and ensemble weight α. The results, reported in Tables 13 and 14, show performance on the LIAR and FakeNewsNet datasets. The sensitivity analysis demonstrates that GETE maintains consistently high performance across a reasonable range of hyperparameter values. In particular, the ensemble weight α converges toward 0.62, confirming its balanced reliance on semantic (Transformer) and relational (GNN) signals. Performance fluctuations remained within ± 0.3%, highlighting the robustness of the framework against hyperparameter variations.

**Table 13:** Sensitivity analysis on LIAR Dataset.

Parameter	Values Tested	Accuracy (%)	F1-score (%)	ROC-AUC (%)
Learning Rate	1e-5/2e-5/3e-5	95.8/96.2/96.0	95.6/96.0/95.9	96.9/97.1/97.0
Batch Size	16/32/64	95.9/96.2/95.8	95.7/96.0/95.6	97.0/97.1/96.8
Ensemble Weight α	0.5/0.62/0.7	95.6/96.2/95.9	95.4/96.0/95.7	96.7/97.1/96.9

**Table 14:** Sensitivity analysis on FakeNewsNet Dataset.

Parameter	Values Tested	Accuracy (%)	F1-score (%)	ROC-AUC (%)
Learning Rate	1e-5/2e-5/3e-5	95.6/96.0/95.9	95.3/95.8/95.6	96.7/97.0/96.9
Batch Size	16/32/64	95.7/96.0/95.7	95.5/95.8/95.5	96.8/97.0/96.7
Ensemble Weight α	0.5/0.62/0.7	95.5/96.0/95.8	95.3/95.8/95.6	96.6/97.0/96.8

### Sensitivity analysis of hyperparameters

To ensure robustness of our framework, we performed a sensitivity analysis on the main hyperparameters of both Transformer and GNN components, namely learning rate, dropout rate, batch size, and training epochs. Learning rate values in the range 1e-5–5e-4 were tested, with stable performance observed around 2e-5–3e-5^10^. Dropout rates between 0.1 and 0.5 showed minor changes (< 2% F1 variation), confirming that moderate dropout helps generalization. Batch sizes from 16 to 128 indicated that smaller batches provide slightly better stability, while larger ones improve efficiency without sacrificing accuracy^5^. Epochs between 5 and 30 demonstrated convergence stabilizing by ~ 15 epochs. Overall, these findings confirm that the selected hyperparameters (learning rate 3e-5, dropout 0.3, batch size 64, 20 epochs) offer a strong balance between stability and efficiency, validating the robustness of our design.

### Comparative evaluation with recent State-of-the-Art models

To further validate GETE, we compared it with several recent baselines, including transformer-only detectors (e.g., BERT/RoBERTa^23^, LLM-based models^10,33^, graph-only methods (e.g., heterogeneous and dynamic GNNs^8,14^, and fusion/ensemble approaches (e.g., multimodal transformers^6,24^, transformer–GCN fusion^21^, and the graph-enhanced ensemble GETAE^31^. Unlike these static-weight fusion methods, our GETE framework employs a meta-learned adaptive weighting mechanism to dynamically balance semantic and relational cues.While transformer-only methods capture strong contextual semantics, they often underperform in modeling relational dependencies, and GNN-only models capture propagation dynamics but lose semantic richness. Fusion and ensemble baselines provide stronger results, yet GETE consistently surpasses them by achieving higher Accuracy, F1, ROC-AUC, and PR-AUC under identical experimental settings. These improvements highlight the advantages of jointly leveraging semantic and relational signals through the meta-learned ensemble mechanism, reinforcing GETE’s robustness and adaptability across diverse fake news scenarios.

### Practical deployment considerations

Beyond accuracy metrics, we also examine the feasibility of deploying the GETE framework in real-time environments such as social media platforms. On our experimental setup with an NVIDIA RTX 3090 GPU, the model achieves an inference throughput of approximately 180–220 articles per second when using batch processing of size 32. This demonstrates the potential for large-scale stream processing in near real-time scenarios. For practical engineering optimizations, techniques such as GPU-accelerated batching, asynchronous data pipelines, and mixed-precision inference can be leveraged to further improve throughput. Additionally, knowledge distillation and model pruning^10,19^ offer promising directions to reduce computational overhead without significantly sacrificing accuracy, enabling deployment on edge devices or cloud-based streaming services. These considerations illustrate that GETE is not only theoretically robust but also adaptable for real-world deployment in dynamic, high-volume social media environments.

### Discussion

The proposed GETE framework yields several important insights into the design of robust misinformation detection systems. First, the meta-learned ensemble mechanism proves effective in adaptively balancing semantic and relational signals, assigning higher weights to transformer-based text encoders when linguistic cues are more discriminative, while leveraging GNN-based relational reasoning when network-level patterns dominate. This flexibility contrasts with prior static hybrid models^23,24^ that fixed fusion weights and thus struggled when dataset characteristics shifted. Second, cross-dataset evaluations highlight GETE’s strong generalization capability. Whereas earlier approaches often demonstrated dataset-specific overfitting^21,32^, GETE adapts effectively to diverse domains, showing that meta-learning can help mitigate domain dependency in fake news detection. This makes the framework particularly promising for deployment in dynamic real-world contexts where news topics, linguistic patterns, and user interactions evolve rapidly. Third, our ablation studies and sensitivity analyses underline the necessity of integrating both semantic and relational modalities. Removing either the transformer or GNN component results in substantial drops in performance, confirming that fake news detection requires complementary modeling of content and propagation. Importantly, the ensemble mechanism ensures robustness against noise in either modality, reducing the risk of failure in cases where text or network signals are incomplete. Fourth, the time performance results suggest that while GETE is computationally more demanding than single-modality models, the trade-off remains favourable. Inference time per document stays within practical limits (< 10 ms), enabling near real-time applications such as automated news triaging, early detection of viral misinformation, or integration into content moderation pipelines. This balance of accuracy and efficiency makes GETE a viable candidate for operational deployment. Nevertheless, some limitations must be acknowledged. GETE’s reliance on relational data means that performance may degrade in scenarios with sparse or unavailable social interaction graphs. Similarly, the joint training of transformer and GNN modules requires substantial GPU resources, which could hinder adoption in low-resource environments. These issues open avenues for future work on lightweight model variants, graph pretraining strategies, and knowledge distillation techniques. Finally, beyond technical performance, our findings emphasize the ethical dimension of deploying automated misinformation detection. Transparency, fairness, and accountability remain essential, as misclassifications could lead to censorship or bias against specific groups. Incorporating interpretability mechanisms and maintaining human-in-the-loop oversight are therefore crucial to ensure responsible use. In summary, GETE not only advances state-of-the-art performance but also contributes to a broader understanding of how semantic and relational modeling can be dynamically integrated for scalable and reliable misinformation detection.

### Limitations

The GETE framework demonstrates strong performance in integrating semantic and relational data but researchers need to identify its multiple existing current restrictions. The model requires user–article–source interaction data to achieve its optimal performance. The GNN module fails to achieve its highest potential when working with text-based datasets including LIAR because relational information stays limited^23–25^. The performance of the model could decrease when working with real-world interaction graphs because these graphs often contain missing data and privacy limitations and noisy information. The model GETE demonstrates universal applicability across LIAR and FakeNewsNet datasets but these datasets contain specific biases which might not represent all types of misinformation that exist in worldwide multilingual environments and specialized domains including health and finance. Our research examined binary classification yet it did not investigate the detailed aspects of multiclass truthfulness labels which include satire and parody and half-truths^21,32^. The training of transformer and GNN modules together as a single model requires additional computational resources because it needs more GPU memory and longer training time compared to single-modality models. The system requires too many resources to function properly so it cannot be used in mobile devices or systems with restricted processing capabilities. AI models need to become more widely used which requires the development of lightweight versions and model compression and distillation methods. The GETE system demonstrates efficient time performance but additional optimization is required for real-time applications to manage extensive social media data streams and active moderation tasks^7,21^. Online or incremental learning mechanisms should be used to manage changing misinformation patterns because they eliminate the need for regular model retraining. The model demonstrates excellent performance on benchmark datasets yet its performance in untested domains and low-resource languages and fast-evolving misinformation scenarios has not been evaluated. Incorporating multilingual transformers, stance detection, and external knowledge graphs could strengthen domain robustness. Ethical and interpretability concerns: Automated misinformation detection raises critical ethical issues, including risks of censorship, overreliance on automated decisions, and potential bias amplification. The interpretability of attention-based visualization faces limitations because more mechanisms need to be added to achieve transparency and fairness and maintain accountability. Human operators need to stay alert for high-risk situations because they stop dangerous misclassification errors from occurring^10^. The system GETE operates as a detection tool which lacks the capability to stop misinformation from spreading independently. The tool requires implementation as part of a complete mitigation strategy which unites warning systems with content authentication protocols and local community response strategies.

### Case study analysis

While CSI, RoBERTa, and BERT incorrectly labeled this article as true due to its coherent and persuasive writing style, our approach successfully detected it as fake by incorporating social-context cues such as atypical propagation. The spread of false information occurs through untrustworthy communication channels and social networks that function beyond established social conventions. A different example of misinformation includes health-related false information about 5G. mobile towers spread COVID-19 among populations.” The baseline models GCN and CSI failed to perform well in this situation. The researcher classified the claim as true because of its repeated appearance in multiple sources although it was not supported by evidence. GETE accurately identified it as fake by combining semantic evidence (scientific implausibility highlighted by BERT) with relational insights (rapid propagation through misinformation-heavy clusters). We studied a verified true headline from FakeNewsNet: “WHO approves new malaria vaccine for widespread use.” Some baselines, particularly GCN, incorrectly marked this as fake due to the sudden spike in sharing activity across unfamiliar nodes. GETE, however, correctly labelled it as true, because it integrated semantic cues (factual medical terminology) with relational evidence (propagation from highly credible, verified sources such as WHO-affiliated accounts). These various examples from political news and scientific reports and sensational news stories show how the integration of relational and semantic information enables GETE to handle nuanced cases that purely text-based or graph-only baselines struggle with. The system demonstrates both the predicted outcome and the explanation behind the decision choice. The GETE system shows its operational abilities through its implementation in actual operational settings.To improve interpretability and gain deeper insights into the model’s decision-making process, we further analyzed the internal attention patterns and graph node importance in representative case studies. For the Transformer component, attention weight visualization was used to identify the most influential words and phrases contributing to classification. We observed that fake news articles tend to have concentrated attention on emotionally charged or sensational keywords such as “breaking,” “shocking,” or “exposed,” whereas real news items exhibit more balanced attention across factual terms and named entities. For the GNN module, node importance scores were computed to reveal which user, article, and source nodes played key roles in influencing predictions. Highly credible sources and well-connected users typically received higher importance values in correctly identified real news cases, while nodes with low connectivity or questionable credibility were more prominent in misclassified examples. Together, these interpretability analyses demonstrate that the GETE framework not only achieves high accuracy but also provides meaningful explanations of its predictions, thereby enhancing transparency and user trust in the system’s outputs.

## Conclusion and future work

In this paper, we introduced GETE, a graph-augmented Transformer ensemble framework designed for robust and scalable fake news detection. By combining the semantic understanding of Transformers with the relational reasoning strengths of GNNs, and integrating them through a meta-learned ensemble, GETE dynamically adapts to dataset characteristics. This adaptive fusion distinguishes it from static weighting strategies employed in existing hybrid approaches. Extensive experiments on the LIAR and FakeNewsNet datasets demonstrated that GETE achieves state of-the-art performance, with significant improvements in accuracy, F1-score, and ROC-AUC over competitive baselines. Ensemble weight analysis further highlighted the complementary roles of semantic and relational features, while ablation studies confirmed the effectiveness of the dynamic weighting strategy. Case studies reinforced these findings, showing how GETE successfully handles subtle cases, such as political misinformation, sensational hoaxes, and health-related rumours, where text-only or graph-only models often fail. The contributions of this work are threefold: (i) introducing a meta-learned ensemble mechanism for adaptive semantic–relational fusion, (ii) validating its robustness and generalization across multiple datasets, and (iii) demonstrating practical strengths through interpretability and case study analysis. Together, these advances provide a step toward more resilient misinformation detection systems. Despite these strengths, the framework has limitations. Training large-scale Transformer and GNN modules jointly requires substantial computational resources. Performance also depends on the quality of user–article–source graph data, which may be incomplete in certain domains. Furthermore, while GETE integrates relational and semantic features effectively, it does not yet address multimodal misinformation that includes images, videos, or audio. Finally, ethical concerns remain around fairness, transparency, and user autonomy, emphasizing the need for human oversight in deployment. While GETE performs well in our experiments, we recognize that running both a Transformer and a GNN together can increase computational load during real-world deployment. Inference remains manageable, but the combined architecture does require more memory and processing power than simpler models. To make GETE even more practical for large-scale or resource-limited environments, future work will explore ways to reduce this overhead such as model compression, pruning, knowledge distillation, and more efficient graph processing. These improvements will help move GETE from being just an effective research model to a lighter, more deployment-friendly solution. Despite the strong performance and generalization ability demonstrated by the GETE framework, several practical challenges remain for real-world deployment. The first challenge is computational efficiency, as training and inference involving both transformer and GNN modules can be resource-intensive on large-scale social media data. Future work can explore model compression, distillation, or sparse attention mechanisms to reduce computational overhead. The second challenge is data privacy and accessibility—social media graphs often include sensitive user interaction data, which may not always be publicly available or ethically usable. Incorporating privacy-preserving learning paradigms, such as differential privacy or federated learning, can help address this concern. Finally, domain drift and evolving misinformation patterns may affect long-term robustness; adaptive retraining strategies and incremental learning pipelines can mitigate this issue. Recognizing and addressing these challenges will facilitate the transition of GETE from research to practical, scalable deployment environments.

## Data Availability

The datasets generated and/or analysed during the current study are available from the corresponding author on reasonable request. “To further support transparency and reproducibility, we have made the source code, dataset splits, and pre-trained models publicly available at: https://github.com/umarfarook2180/Fake-News-Detector.
